# A Seven-microRNA Expression Signature Predicts Survival in Hepatocellular Carcinoma

**DOI:** 10.1371/journal.pone.0128628

**Published:** 2015-06-05

**Authors:** Jian Zhang, Charing C. N. Chong, George G. Chen, Paul B. S. Lai

**Affiliations:** Department of Surgery, The Chinese University of Hong Kong, Prince of Wales Hospital, Shatin, New Territories, Hong Kong SAR, China; The University of Hong Kong, CHINA

## Abstract

Hepatocellular carcinoma (HCC) is the fifth common cancer. The differential expression of microRNAs (miRNAs) has been associated with the prognosis of various cancers. However, limited information is available regarding genome-wide miRNA expression profiles in HCC to generate a tumor-specific miRNA signature of prognostic values. In this study, the miRNA profiles in 327 HCC patients, including 327 tumor and 43 adjacent non-tumor tissues, from The Cancer Genome Atlas (TCGA) Liver hepatocellular carcinoma (LIHC) were analyzed. The associations of the differentially expressed miRNAs with patient survival and other clinical characteristics were examined with t-test and Cox proportional regression model. Finally, a tumor-specific miRNA signature was generated and examined with Kaplan–Meier survival, univariate\multivariate Cox regression analyses and KEGG pathway analysis. Results showed that a total of 207 miRNAs were found differentially expressed between tumor and adjacent non-tumor HCC tissues. 78 of them were also discriminatively expressed with gender, race, tumor grade and AJCC tumor stage. Seven miRNAs were significantly associated with survival (P value <0.001). Among the seven significant miRNAs, six (hsa-mir-326, hsa-mir-3677, hsa-mir-511-1, hsa-mir-511-2, hsa-mir-9-1, and hsa-mir-9-2) were negatively associated with overall survival (OS), while the remaining one (hsa-mir-30d) was positively correlated. A tumor-specific 7-miRNAs signature was generated and validated as an independent prognostic predictor. Collectively, we have identified and validated an independent prognostic model based on the expression of seven miRNAs, which can be used to assess patients’ survival. Additional work is needed to translate our model into clinical practice.

## Introduction

Liver cancer is the fifth most common cancer in men and the second cause of cancer death worldwide (cause 745 000 deaths in 2012) [1]. Hepatocellular carcinoma (HCC) is the most common type of liver cancers, accounting for about 75% of all liver cancer cases [2]. The majority of HCC patients are diagnosed at an advanced stage. It is important to discover tumor-specific prognostic factors for HCC to foresee outcome and improve treatments. To date, microRNAs (miRNAs) have been proposed as a promising prognostic predictor in HCC [3].

miRNAs play vital roles in mediating the expression of proteins by regulating the transcription or degradation of target mRNAs [4]. In HCC, a number of miRNAs have been associated with survival or response to chemotherapy such as sorafenib or doxorubicin [5–7]. However, the sample size, the numbers of candidate miRNAs or miRNA detection method in previous studies were relatively limited [8–10].

TCGA project provides a collection of clinical data, RNA sequence, DNA methylation, DNA copy number variations, and miRNA sequence profiles for LIHC. Here, we designed a study using the dataset from TCGA project: 1) to find out the differential miRNAs expression profiles between HCC tumors and adjacent non-tumor tissues, 2) to determine the miRNAs with prognostic potential from the differential expression profiles, and 3) to understanding the biological pathway of the prognostic miRNA signature.

## Materials and Methods

### Patients and samples

All 360 patients of LIHC were retrieved from the TCGA data portal. The full clinical dataset were downloaded (up to Feb 24, 2015) and checked for the assessment of the eligibility. The exclusion criteria were set as follows: 1) histologic diagnosis is not HCC, including fibrolamellar carcinoma (2 cases) and mixed hepatocholangiocarcinoma (7 cases); 2) samples with clinical data but without miRNA sequence data (4 cases); and 3) Overall survival more than 2000 days or missing important clinical or biological data (20 cases). Overall, a total of 327 HCC patients were included in our study with the corresponding clinical data including age, gender, race, the American Joint Committee on Cancer staging system (also be called AJCC TNM staging system), tumor status, vital status, risk factors, vascular invasion, Child-Pugh classification and surgical types. Among the 327 patients (Cohort T), the adjacent non-tumor tissues were retrieved from 43 subjects (Cohort N). As the data were obtained from TCGA, further approval by an ethics committee was not required. This study meets the publication guidelines provided by TCGA (http://cancergenome.nih.gov/publications/publicationguidelines).

### MiRNA expression data procession

The miRNA expression data (level 3) of the corresponding patients (tumor and/or adjacent non-tumor tissues) were downloaded from TCGA data portal (up to Feb 24, 2015). The miRNA expression profiling was performed using the Illumina HiSeq 2000 miRNA sequencing platforms (Illumina Inc, San Diego, CA). The miRNA expression level was demonstrated as reads per million miRNA mapped data. The miRNA expression analyses were performed using BRB-ArrayTools (version 4.4) developed by Dr. Richard Simon and the BRB-ArrayTools Development Team [11]. In brief, the miRNAs with missing data exceeded 10% of all subjects were excluded from the dataset and the expression level of each individual miRNA was log2-transformed for further analysis.

### KEGG Pathway Analysis of miRNA Target Genes

We used DIANA-mirPath [12] for the KEEG pathway analysis of miRNA signature. DIANA-miRPath is a miRNA pathway analysis web-server and utilize predicted miRNA targets provided by the DIANA-microT-CDS algorithm. Using default settings, DIANA-miPath unions all the miRNA target genes and computes P values that describe KEGG pathway enrichment of the target genes.

### Statistical analysis

The continuous variables were presented as mean ± SD. The differences of clinical characteristics (age, gender, risk factors, AJCC stage, tumor size, lymph node involvement, Metastasis status, and Tumor grade) between two cohorts (Cohort N and Cohort T) were evaluated using Chi-square tests.

The miRNAs expression levels between tumor and adjacent non-tumor tissues were ascertained with two-sample t test, and the significance level was set as 0.001 as default to control the false discovery rate (FDR). Subsequently, the univariate Cox proportional hazards regression was conducted to find out the miRNAs correlated with overall survival [13]. After selecting the miRNAs, the principal component model was applied to compute a prognostic index for each patient.

Kaplan–Meier survival analysis and univariate/multivariate Cox proportional hazards regression analyses were carried out to compare various groups of miRNAs signature (high vs. low risk), gender (Female vs. Male), race (White vs. Asian), tumor status (With tumor vs Tumor free), tumor grade (G3+G4 vs. G1+G2), AJCC TNM stage system (tumor size, T3+T4 vs. T1+T2), AJCC pathological stage system (stage III + IV vs. stage I + II), and age at diagnosis (≥60 vs. <60). P value less than 0.05 was considered as statistical unless specifically indicated. The statistical analyses were performed by BRB-ArrayTools and SPSS 20 (IBM, USA).

## Results

### Patient characteristics

All 327 patients enrolled in our study were pathologically diagnosed with HCC. The mean ± SD age for all 327 patients was 60.37 ± 16.71 years, and the mean ± SD follow-up time was 17.16 ± 17.75 months. Among the 327 participants (Cohort T), a total of 43 patients with adjacent non-tumor tissues were included in Cohort N for the analysis of the differential expression of miRNAs between tumor and adjacent non-tumor tissues. As summarized in Table 1, no significant difference was observed in the distribution of age (P = 0.857), gender (P = 0.402), risk factors (P = 0.694), AJCC pathological stage (P = 0.235), tumor size (P = 0.901), lymph node involvement (P = 0.480), Metastasis status (P = 0.461), and Tumor grade (P = 0.168) between the two cohorts.

**Table 1:** Clinical characteristics of patients with hepatocellular cell carcinoma.

Category	Cohort N (n = 43)	Cohort T (n = 327)	P value ^a^
Age, mean ± SD	60.37 ± 16.71	59.89 ± 13.00	0.857
Gender			0.402
Male	27	226	
Female	16	101	
Race			**0.000**
Asian	6	153	
African	6	15	
White	29	149	
NA	2	10	
Tumor status			**0.000**
Tumor free	18	211	
With tumor	18	95	
NA	7	21	
Vital status			**0.000**
Alive	18	249	
Dead	25	78	
Risk factor			0.694
Yes	27	236	
No	9	67	
NA	7	24	
AJCC pathological stage			0.235
Stage I	15	150	
Stage II	9	75	
Stage III	11	75	
Stage IV	1	6	
NA	7	21	
AJCC TNM staging system			
Tumor size			0.901
T1	16	159	
T2	12	82	
T3	12	70	
T4	3	13	
TX	0	3	
Lymph node involvement			0.480
N0	27	227	
N1	1	4	
NX	14	95	
NA	1	1	
Metastasis status			0.461
M0	28	243	
M1	1	4	
MX	14	81	
Vascular invasion			**0.008**
Macro	3	15	
Micro	8	84	
No	26	178	
NA	6	50	
Tumor grade			0.168
G1	3	42	
G2	23	155	
G3	15	114	
G4	0	12	
NA	2	4	
Child-Pugh classification			**0.000**
A	20	195	
B	8	18	
C	0	1	
NA	15	111	
New tumor event			**0.000**
No	1	161	
Yes	0	89	
NA	47	96	

AJCC: American Joint Committee on Cancer; NA: Not Available; Risk factors: Alcohol consumption, Hepatitis B/C, Hemochromatosis, Nonalcoholic Fatty Liver Disease, Alpha-1 Antitrypsin Deficiency

^a^ Statistical significant results (in bold)

### Differentially expressed miRNAs (DEmiRNA) in tumor and adjacent non-tumor tissues

**Fig 1:**
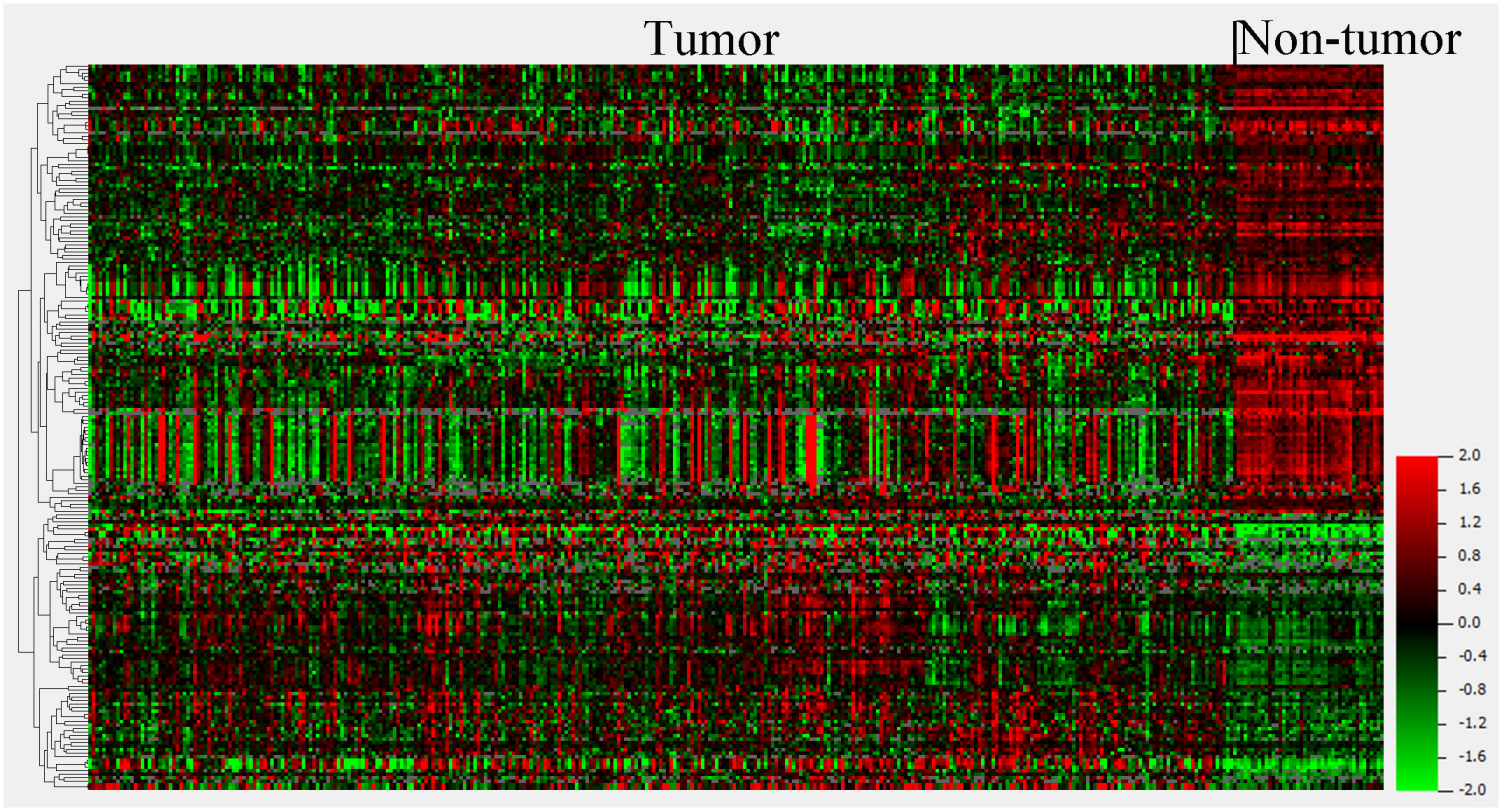
Heatmap of 207 differentially expressed miRNAs in tumor/non-tumor. The miRNA expression levels are mean-centered across samples, and an unsupervised hierarchical clustering with complete linkage is carried out by Cluster 3.0.

**Fig 2:**
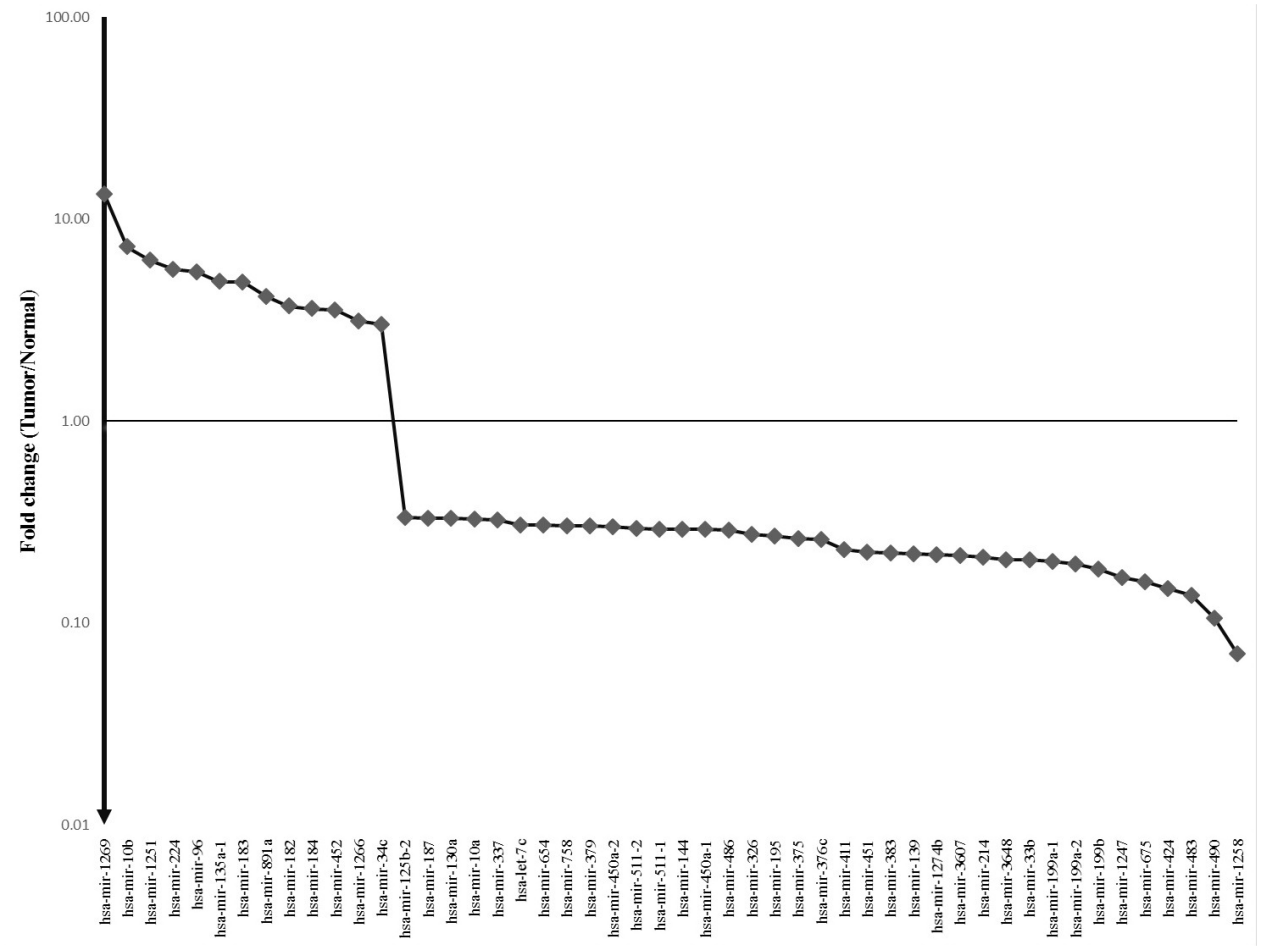
Fifty differentially expressed miRNAs (Absolute fold changes >3). The expression levels of the significant miRNAs in hepatocellular cell carcinoma (HCC) are compared to those of adjacent non-tumor liver tissue, as fold change (tumor vs. non-tumor).

The miRNA expression in the 327 HCC tumor tissues and 43 adjacent non-tumor tissues (Cohort T and N) were investigated, and a total of 207 miRNAs (DEmiRNA) were found to be expressed differentially with p value and FDR less than 0.001 (S1 Table). As outlined in Fig 1, the unsupervised hierarchical clustering with the 207 miRNA expression data clearly discriminated the tumor and adjacent non-tumor samples. Among these 207 miRNAs, 77 miRNAs (31.2%) were up-regulated, while the remaining 130 miRNAs (62.8%) were down-regulated. With regard to the fold change in expression levels, 50 differentially expressed miRNAs showed a greater than three-fold or less than 0.33-fold change in expression levels. Among the 50 miRNAs, 13 miRNAs (26.0%) were up-regulated and 37 miRNAs (74.0%) were down-regulated (Fig 2).

### The correlation between differentially expressed miRNAs from Tumor/Non-tumor and clinical parameters

The 207 DEmiRNA sets from Tumor and Non-tumor HCC tissues were further analyzed according to clinical parameters including gender, race, tumor status, risk factors, tumor grade, AJCC pathologic (TNM stage), AJCC tumor stage, vascular invasion, Child-Pugh classification, and age at diagnosis.

A total of 78 miRNAs were found to be expressed differentially with P value less than 0.001 (S2 Table). Most of these miRNAs are unevenly expressed in gender, race, tumor grade and AJCC pathological stage; No significant miRNA was found between different tumor status, AJCC TNM staging system (lymph node), vascular invasion and Child-Pugh classification (Table 2).

**Table 2:** Differentially expressed miRNAs from Tumor/Non-tumor according to clinical parameters.

Comparison	Down-regulated	Up-regulated	Total
Gender (Female vs. Male)	4 (26.7%)	11 (73.3%)	15 (19.2%)
Race (White vs. Asian)	5 (33.3%)	10 (66.7%)	15 (4.3%)
Risk factors (No vs. Yes)	0 (0.0%)	1 (100%)	1 (0.3%)
Tumor grade (G3+G4 vs. G1+G2)	9 (56.3%)	7 (43.8%)	16 (4.6%)
AJCC TNM staging system			
T (T3+T4 vs. T1+T2)	3 (37.5%)	5 (62.5%)	8 (2.3%)
N (N1 vs. N0)	1 (50.0%)	1 (50.0%)	2 (0.6%)
AJCC pathological stage (III + IV vs. I + II)	3 (27.3%)	8 (72.7%)	11 (3.2%)
New tumor event (Yes vs. No)	0 (0.0%)	2 (100%)	2 (0.6%)
Age at diagnosis (≥60 vs. <60)	4 (50.0%)	4 (50.0%)	8 (2.3%)

AJCC American Joint Committee on Cancer; Tumor grade: neoplasm histologic grade; vs. versus.

### Establishment of miRNAs signature associated with HCC patient survival

**Fig 3:**
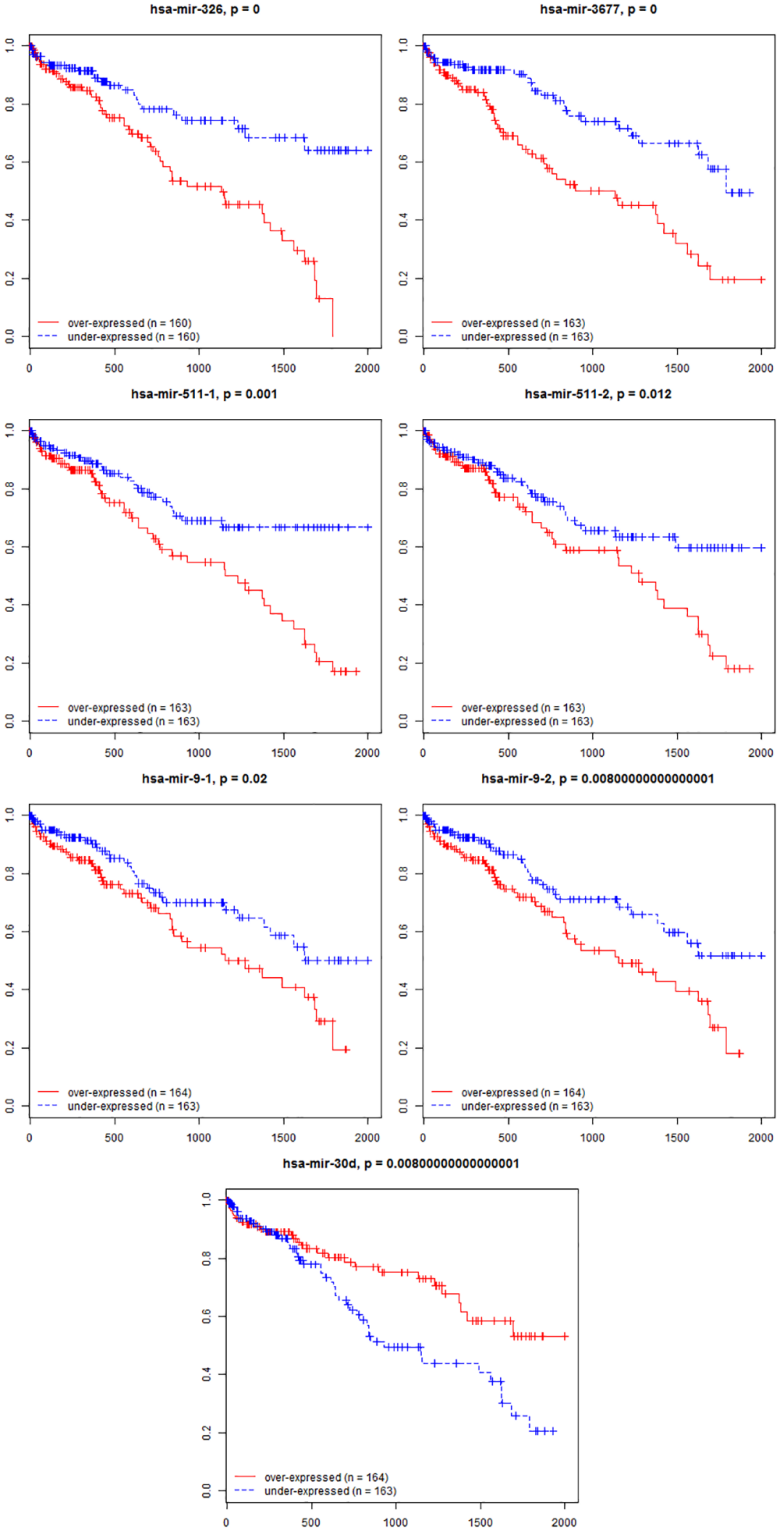
Kaplan-Meier survival curves for 7-miRNA signature. Six miRNA (hsa-mir-326, hsa-mir-3677, hsa-mir-511-1, hsa-mir-511-2, hsa-mir-9-1, and hsa-mir-9-2) were negatively associated with OS and one (hsa-mir-30d) was positively correlated. (Red line: overexpressed, Blue line: under-expressed, Horizontal axis: overall survival time, Vertical axis: survival function).

To identify the potential miRNAs with prognostic characteristics, the 207 DEmiRNA expression in the 327 HCC tumor tissues (Cohort T) was profiled using the univariate Cox proportional hazards regression model and seven miRNAs were found to be significantly associated with survival (P <0.001, S3 Table). Among the seven significant miRNAs, six (hsa-mir-326, hsa-mir-3677, hsa-mir-511-1, hsa-mir-511-2, hsa-mir-9-1, and hsa-mir-9-2) were negatively associated with OS, while the remaining one (hsa-mir-30d) was positively correlated (Fig 3).

Then, a principal component model was used to compute a prognostic index for each patient by Cox proportional hazards models and Leave-One-Out-Cross-Validation. The prognostic index can be computed by the simple formula Σiwi xi—1.880964 where wi and xi are the weight and logged miRNA expression for the i-th miRNA. A patient was predicted as high (low) risk if its prognostic index is larger than (smaller than or equal to) -0.05488. Accordingly, cohort T can be divided into two groups: high-risk (n = 161) and low-risk (n = 166) groups.

### Associations of the 7-miRNA signature model with clinical parameters

Univariate and multivariate Cox regression analyses were used to test the effect of the 7-miRNA signature (high versus low risk) on overall survival taking into account the following demographic and clinical parameters: (i) gender (Female vs. Male); (ii) tumor status (With tumor vs Tumor free); (iii) tumor grade (G3+G4 vs. G1+G2); (iv) AJCC TNM staging system (T) (T3+T4 vs. T1+T2); (v) and AJCC pathological stage (III–IV versus I–II).

**Fig 4:**
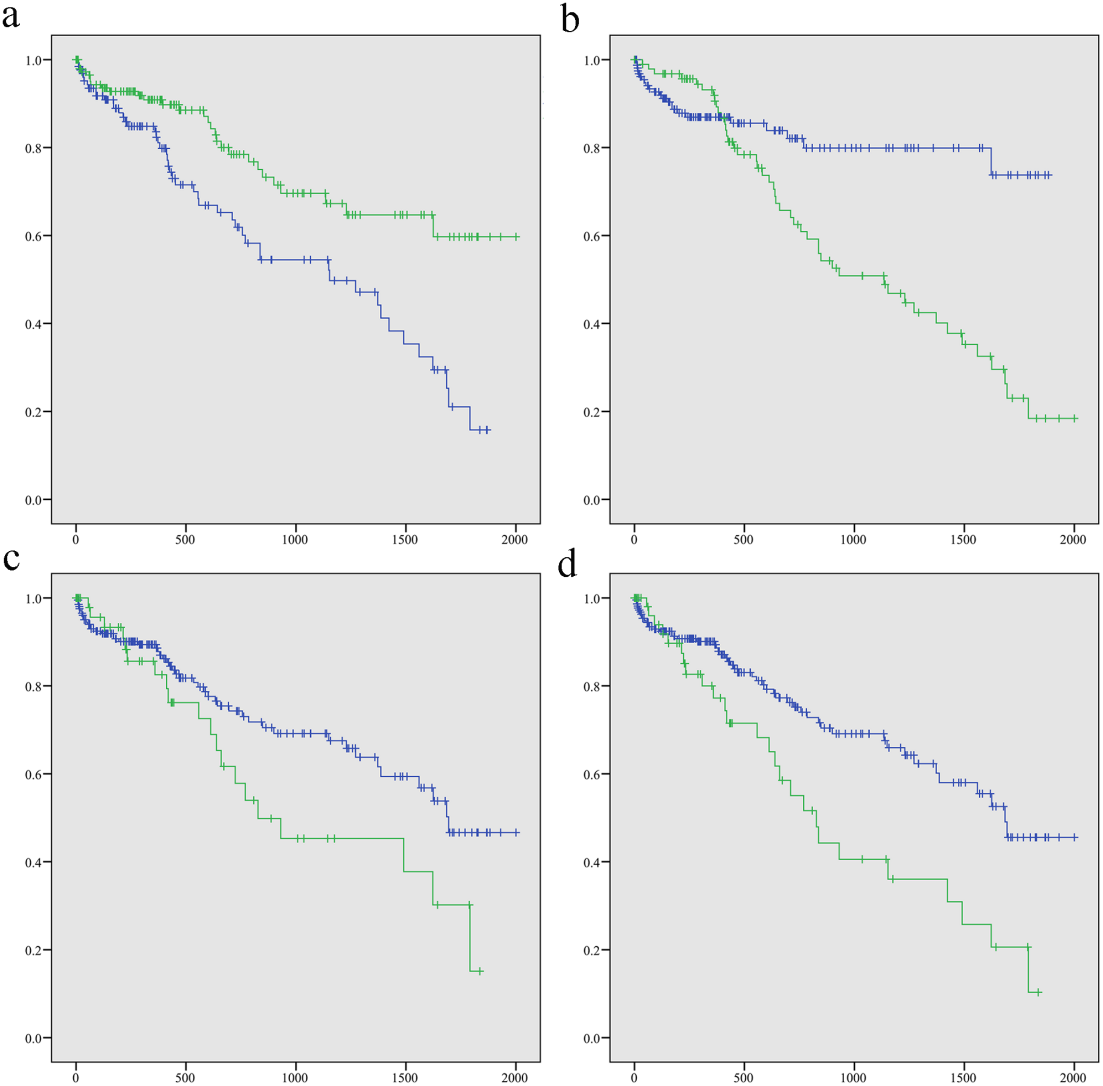
Kaplan–Meier survival curves for hepatocellular cell carcinoma patients. The 327 hepatocellular cell carcinoma patients are compared in two groups according to: a 7-miRNA signature (1 high risk vs. 2 low risk); b Tumor status (1 Tumor free vs. 2 With tumor); c AJCC pathological (1 I + II vs. 2 stage III + IV); and d AJCC T stage (1 T1+T2 vs. 2 T3+T4). Log Rank (Mantel-Cox) P value are 0.000, 0.001, 0.042 and 0.002, respectively. (Blue line: group 1, Green line: group 2, Horizontal axis: overall survival time, Vertical axis: survival function).

As summarized in Table 3 and Fig 4, in univariate analysis, 7-miRNA signature, tumor status, AJCC T stage, and AJCC pathological stage rather than gender and tumor grade were associated with patients’ OS. In multivariate analysis, the risk established by the 7-miRNA model and tumor status resulted to be as an independent prognostic factor after the final stepwise analysis (P < 0.01).

**Table 3:** Univariate and multivariate analysis of parameters associated with overall survival.

		Univariate analysis		Multivariate analysis	
		HR(95% CI)	P value ^a^	HR(95% CI)	P value ^a^
7-miRNA signature	High vs. low risk	2.22 (1.40–3.52)	**0.001**	2.06 (1.21–3.52)	**0.008**
Gender	Female vs. Male	1.40 (0.88–2.23)	0.155	1.34 (0.78–2.30)	0.288
Tumor status	With tumor vs. Tumor free	2.33 (1.41–3.85)	**0.001**	2.06 (1.21–3.51)	**0.008**
Tumor grade	G3+G4 vs. G1+G2	1.14 (0.721–1.79)	0.584	1.18 (0.70–2.00)	0.533
AJCC TNM staging system (T)	T3+T4 vs. T1+T2	2.11 (1.32–3.37)	**0.002**	2.34 (0.30–17.98)	0.415
AJCC pathological stage	III + IV vs. I + II	1.70 (1.01–2.87)	**0.045**	0.62 (0.08–4.65)	0.642

AJCC American Joint Committee on Cancer; Tumor grade: neoplasm histologic grade; HR hazard ratio, CI confidential interval; vs. versus.

^a^ Statistical significant results (in bold)

### KEGG pathway analysis for 7-miRNA signature target genes

**Fig 5:**
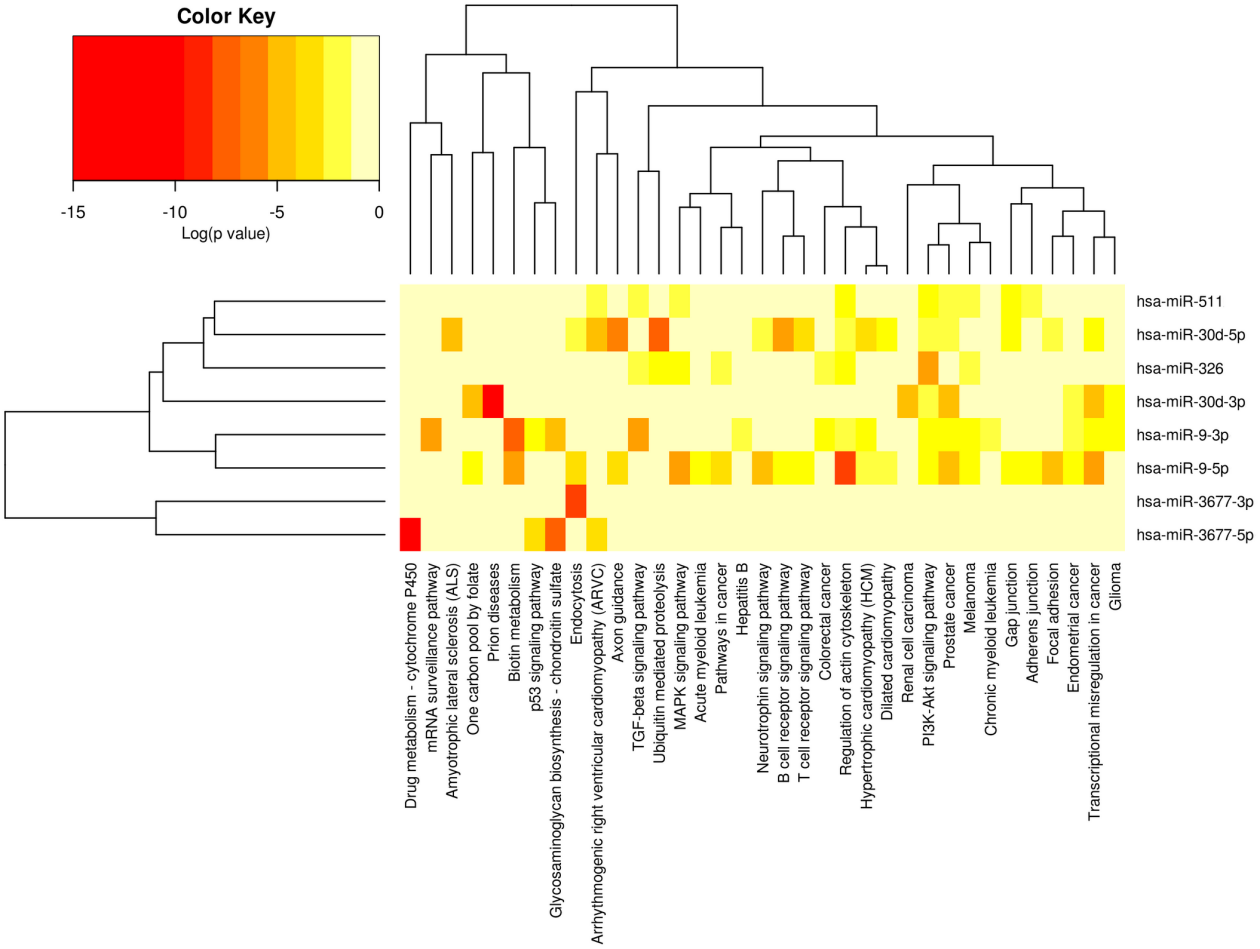
Heatmap of KEGG pathways enriched in seven miRNA target genes. The isoforms of 7-miRNA signature were involved in multiple pathways, especially cancer-specific pathways. (DIANA-mirpath computes log_10_ P-values).

The miRNAs selected in our study were correlated with several cancers and diseases by previous studies [14–16]. However, we check whether any KEGG pathways were enriched with the seven miRNAs target genes to reveal the biological relevance. The seven miRNAs target genes participated in both cancer-related and non-cancer related pathways (Fig 5).

We listed the target-gene enrichment of KEGG pathways in our study (Table 4 and S4 Table). Ten cancer-related pathways including pathways for prostate cancer, colorectal cancer, endometrial cancer, acute myeloid leukemia, melanoma, and renal cell carcinoma, were enriched with the seven miRNA target genes. Another 25 non-cancer related pathways involved in prion diseases, cardiomyopathy, Hepatitis B, drug metabolism, endocytosis, P53 signaling pathway, and PI3K-Akt signaling pathway et al were also enriched with the seven miRNA target genes. Thus, KEEG pathway analysis of signature microRNAs has identified potential targets and biological processes known to be involved in cancer which provided the biological relevance of the 7-microRNA signature.

**Table 4:** Cancer related and non-cancer related KEGG pathways enriched in seven miRNA target genes. (The full list of target genes see S4 Table)

	KEGG pathway	Number of miRNAs	Number of genes	P-value
Cancer related pathways	Transcriptional misregulation in cancer	4	45	<0.001
	Prostate cancer	5	30	<0.001
	Melanoma	4	21	<0.001
	Pathways in cancer	2	38	0.001
	Colorectal cancer	2	10	0.003
	Acute myeloid leukemia	1	8	0.003
	Endometrial cancer	3	12	0.004
	Renal cell carcinoma	1	5	0.005
	Chronic myeloid leukemia	1	6	0.035
	Glioma	2	9	0.012
Non-cancer related pathways	Prion diseases	1	1	<1e-16
	Regulation of actin cytoskeleton	5	65	<0.001
	PI3K-Akt signaling pathway	6	88	<0.001
	Endocytosis	3	34	<0.001
	Glycosaminoglycan biosynthesis—chondroitin sulfate	2	2	<0.001
	Biotin metabolism	2	1	<0.001
	MAPK signaling pathway	3	45	<0.001
	Ubiquitin mediated proteolysis	2	28	<0.001
	Axon guidance	2	31	<0.001
	Arrhythmogenic right ventricular cardiomyopathy (ARVC)	3	15	<0.001
	Drug metabolism—cytochrome P450	1	1	<0.001
	TGF-beta signaling pathway	3	18	<0.001
	Focal adhesion	2	33	<0.001
	B cell receptor signaling pathway	2	16	<0.001
	Gap junction	3	19	<0.001
	Hypertrophic cardiomyopathy (HCM)	3	21	<0.001
	mRNA surveillance pathway	1	10	0.003
	Dilated cardiomyopathy	2	17	0.003
	Neurotrophin signaling pathway	2	23	0.004
	One carbon pool by folate	2	6	0.004
	p53 signaling pathway	2	9	0.008
	T cell receptor signaling pathway	2	20	0.009
	Adherens junction	2	15	0.017
	Amyotrophic lateral sclerosis (ALS)	1	9	0.034
	Hepatitis B	1	10	0.044

## Discussion

Computational analysis of TCGA dataset has been demonstrated to be a powerful approach in identifying genetic and transcriptional changes linked to clinical outcomes, pointing the way to new prognostic markers and potentially novel therapeutic targets [14, 17, 18]. The identification and validation of novel biomarkers such as miRNA signatures have been a research focus in cancer research [3]. In the present study, we identified a tumor-specific miRNA signature consisting of seven miRNAs that were significantly associated with survival in patients with HCC, on the basis of genome-wide miRNA profiling of 327 HCC patients. Furthermore, we confirmed the 7-miRNA signature as an independent prognostic factor after adjusting to the various variables including gender, tumor status, tumor grade, AJCC T stage and AJCC pathological stage. The performance of our generated prognostic model was assessed in Leave-one-out cross-validation (LOOCV) model to refine its accuracy.

As a subset of non-coding RNAs, mounting evidences have indicated that the miRNA function as an important regulator in the development and progression of HCC, including cell migration [19], apoptosis [20], and response to antitumor therapies [21]. A number of miRNAs have been correlated with the survival of HCC patients [22] and thus this is not the first study aimed to establish a prognostic signature in HCC. In fact, Budhu et al. aiming at identifying biological meaningful metastasis-related miRNAs, reported a 20-miRNA metastasis signature that predicted HCC with venous metastases and was associated with survival [8]. Another report, Jiang et al. found a 19-miRNA signature that impacted HCC outcome [9]. These two reports found some miRNAs that also be used in our analysis. However, the numbers of candidate miRNAs, miRNA detection methods and goals of previous studies are different from this study; in particular, our approach has two major advantages: 1) We use novel stepwise approach that integrating TCGA dataset with 327 HCC tumor tissues and 43 adjacent non-tumor tissues, which is by far the largest research population for HCC survival prediction; 2) instead of using miRNA arrays (about 500 candidate miRNAs) or real-time PCR (about 100 candidate miRNAs), TCGA dataset was collected from high-thorough miRNA expression profiling using the Illumina HiSeq 2000 miRNA Sequencing platforms (1246 candidate miRNAs) which makes it possible to include as many miRNAs as we can.

Hence, we conducted the study to identify a tumor-specific miRNA signature in a large cohort of HCC patients with Next Generation Sequencing technology. First of all, a summary of 207 miRNAs were found to be expressed differentially between tumor and adjacent non-tumor tissues. Seventy-eight of the 207 miRNAs were also differentially expressed according to gender, race, tumor grade, AJCC TNM stage, age at diagnosis and AJCC tumor stage. Subsequently, the expression levels of seven miRNAs were demonstrated to be significantly associated with the prognosis of HCC patients, and a 7-miRNA signature was generated and confirmed as an independent prognostic factor. Finally, the KEGG pathway analysis proved that the 7-miRNA signature is biologically meaningful. With the largest cohort of HCC patients and candidate miRNAs, this is by far the most integrated study to elucidate the miRNA expression profiles in HCC and evaluate their prognostic values.

With respect to the associations between their expression levels and patient survival, the seven miRNAs in the signature model were divided into two groups: six risky miRNAs that were negatively associated with patient survival and the remaining one protective miRNAs positively associated. Among the six risky miRNAs, the methylation of hsa-mir-9-1 was observed in liver fibrolamellar carcinoma [23], Burkitt lymphoma [24], pancreatic adenocarcinoma [25], and breast cancer [26]. Aberrant hypermethylation-mediated inactivation of has-mir-9-1 is known to prolong the overall survival in metastasized renal cell cancer [27]. The methylation of has-mir-9-2 is also a frequent event in human HCC [28]. Expression of hsa-miR-326 was correlated with poor prognosis in esophageal cancer and glioma patients [29, 30]. Hsa-miR-511 impeded HCC cell proliferation, migration, and invasion by targeting PIK3R3 [31]. Meanwhile, the functions of mir-3677 in cancer was poorly understood. The biological and clinical studies of these miRNAs have partially provided clues for the prognostic value, but we need well-designed studies to validate the functions of these miRNAs in HCC.

Regarding the protective miRNA, Yao et al. [32] reported that hsa-mir-30d was up-regulated in HCC and promoted HCC cell invasion and metastasis, which is consistent with our finding, but the prognostic characteristics of hsa-mir-30d in HCC remains to be clarified. Higher expression of hsa-miR-30d was associated with better overall survival or inhibition of tumor cell in renal carcinoma [33], ovarian carcinoma [34] and malignant peripheral nerve sheath tumor [35]. Hsa-miR-30d was involved in cell autophagy [36], apoptosis [37] and platinum sensitivity in tumor cells [38]. The biological studies certified that hsa-mir-30d had tumor-suppressive character, which was also consistent with our finding.

There are some limitations should be acknowledged in interpreting the above results. First, the miRNA expression profiling was detected from liver tissues, which may not reflect the miRNA in serum or stool [39]. We may need to validate the miRNA signature in these samples as they are conveniently available for monitoring. Second, we strictly selected the subjects and use the LOOCV model to cross-validate the 7-miRNA prognostic model, we still need external validation to reduce the FDR.

In conclusion, by employing a large independent HCC patient cohort, our study identified a tumor-specific miRNA signature consisting of seven miRNAs, which can be served as a novel biomarker for HCC prognostic prediction and improve treatment outcome.

## Supporting Information

S1 Table Summary of miRNAs expressed differentially between tumor and adjacent non-tumor tissues (DOCX)

S2 Table Differentially expressed miRNAs from Tumor/Non-tumor according to clinical parameters. (DOCX)

S3 Table miRNAs associated with overall survival by univariate Cox regression analysis. (DOCX)

S4 Table Cancer related and non-cancer related KEGG pathways enriched in seven miRNA target genes (DOCX)

## References

[1] Stewart BW, Wild CP. World Cancer Report 2014 Lyon CEDEX, France: The International Agency for Research on Cancer (IARC).

[2] Ahmed I, Lobo DN. Malignant tumours of the liver. Surgery (Oxford) 2009; 27: 30–37. 10.1016/j.mpsur.2008.12.005

[3] Villanueva A, Hoshida Y, Toffanin S, Lachenmayer A, Alsinet C, Savic R, et al New strategies in hepatocellular carcinoma: genomic prognostic markers. Clin Cancer Res 2010; 16: 4688–4694. 10.1158/1078-0432.CCR-09-1811 20713493 PMC3395071

[4] Lu J, Getz G, Miska EA, Alvarez-Saavedra E, Lamb J, Peck D, et al MicroRNA expression profiles classify human cancers. Nature 2005; 435: 834–838. 10.1038/nature03702 15944708

[5] Zhang J, Wang Y, Zhen P, Luo X, Zhang C, Zhou L, et al Genome-wide analysis of miRNA signature differentially expressed in doxorubicin-resistant and parental human hepatocellular carcinoma cell lines. PLoS One 2013; 8: e54111 10.1371/journal.pone.0054111 23359607 PMC3554743

[6] Vaira V, Roncalli M, Carnaghi C, Faversani A, Maggioni M, Augello C, et al MicroRNA-425-3p predicts response to sorafenib therapy in patients with hepatocellular carcinoma. Liver Int 2015; 35: 1077–1086. 10.1111/liv.12636 25040368

[7] Wang Y, Gao X, Wei F, Zhang X, Yu J, Zhao H, et al Diagnostic and prognostic value of circulating miR-21 for cancer: a systematic review and meta-analysis. Gene 2014; 533: 389–397. 10.1016/j.gene.2013.09.038 24076132

[8] Budhu A, Jia HL, Forgues M, Liu CG, Goldstein D, Lam A, et al Identification of metastasis-related microRNAs in hepatocellular carcinoma. Hepatology 2008; 47: 897–907. 10.1002/hep.22160 18176954

[9] Jiang J, Gusev Y, Aderca I, Mettler TA, Nagorney DM, Brackett DJ, et al Association of MicroRNA expression in hepatocellular carcinomas with hepatitis infection, cirrhosis, and patient survival. Clin Cancer Res 2008; 14: 419–427. 10.1158/1078-0432.CCR-07-0523 18223217 PMC2755230

[10] Barry CT, D'Souza M, McCall M, Safadjou S, Ryan C, Kashyap R, et al Micro RNA expression profiles as adjunctive data to assess the risk of hepatocellular carcinoma recurrence after liver transplantation. Am J Transplant 2012; 12: 428–437. 10.1111/j.1600-6143.2011.03788.x 22008552

[11] Zhao Y, Simon R. BRB-ArrayTools Data Archive for human cancer gene expression: a unique and efficient data sharing resource. Cancer Inform 2008; 6: 9–15. 19259398 10.4137/cin.s448 PMC2623314

[12] Papadopoulos GL, Alexiou P, Maragkakis M, Reczko M, Hatzigeorgiou AG. DIANA-mirPath: Integrating human and mouse microRNAs in pathways. Bioinformatics 2009; 25: 1991–1993. 10.1093/bioinformatics/btp299 19435746

[13] Bair E, Tibshirani R. Semi-supervised methods to predict patient survival from gene expression data. PLoS Biol 2004; 2: E108 10.1371/journal.pbio.0020108 15094809 PMC387275

[14] Yan W, Li R, Liu Y, Yang P, Wang Z, Zhang C, et al MicroRNA expression patterns in the malignant progression of gliomas and a 5-microRNA signature for prognosis. Oncotarget 2014; 5: 12908–12915. 25415048 10.18632/oncotarget.2679 PMC4350338

[15] Ge YZ, Wu R, Xin H, Zhu M, Lu TZ, Liu H, et al A tumor-specific microRNA signature predicts survival in clear cell renal cell carcinoma. J Cancer Res Clin Oncol. 2015 (in press) 10.1007/s00432-015-1927-0 PMC11824131 25633718

[16] Fleming NH, Zhong J, da Silva IP, Vega-Saenz de Miera E, Brady B, Han SW, et al Serum-based miRNAs in the prediction and detection of recurrence in melanoma patients. Cancer 2015; 121: 51–59. 10.1002/cncr.28981 25155861 PMC4270907

[17] Gu Y, Zhang M, Peng F, Fang L, Zhang Y, Liang H, et al The BRCA1/2-directed miRNA signature predicts a good prognosis in ovarian cancer patients with wild-type BRCA1/2. Oncotarget 2015; 6: 2397–2406. 25537514 10.18632/oncotarget.2963 PMC4385859

[18] Barbano R, Palumbo O, Pasculli B, Galasso M, Volinia S, D'Angelo V, et al A miRNA signature for defining aggressive phenotype and prognosis in gliomas. PLoS One 2014; 9: e108950 10.1371/journal.pone.0108950 25279461 PMC4184816

[19] Wong QW, Ching AK, Chan AW, Choy KW, To KF, Lai PB, et al MiR-222 overexpression confers cell migratory advantages in hepatocellular carcinoma through enhancing AKT signaling. Clin Cancer Res 2010; 16: 867–875. 10.1158/1078-0432.CCR-09-1840 20103675

[20] Li J, Fu H, Xu C, Tie Y, Xing R, Zhu J, et al miR-183 inhibits TGF-beta1-induced apoptosis by downregulation of PDCD4 expression in human hepatocellular carcinoma cells. BMC Cancer 2010; 10: 354 10.1186/1471-2407-10-354 20602797 PMC2909210

[21] Zhang G, Wang Q, Xu R. Therapeutics Based on microRNA: A New Approach for Liver Cancer. Curr Genomics 2010; 11: 311–325. 10.2174/138920210791616671 21286309 PMC2944997

[22] Yang N, Ekanem NR, Sakyi CA, Ray SD. Hepatocellular carcinoma and microRNA: new perspectives on therapeutics and diagnostics. Adv Drug Deliv Rev 2015; 81: 62–74. 10.1016/j.addr.2014.10.029 25450260

[23] Trankenschuh W, Puls F, Christgen M, Albat C, Heim A, Poczkaj J, et al Frequent and distinct aberrations of DNA methylation patterns in fibrolamellar carcinoma of the liver. PLoS One 2010; 5: e13688 10.1371/journal.pone.0013688 21060828 PMC2966398

[24] Onnis A, De Falco G, Antonicelli G, Onorati M, Bellan C, Sherman O, et al Alteration of microRNAs regulated by c-Myc in Burkitt lymphoma. PLoS One 2010; 5: e12960 10.1371/journal.pone.0012960 20930934 PMC2945769

[25] Omura N, Li CP, Li A, Hong SM, Walter K, Jimeno A, et al Genome-wide profiling of methylated promoters in pancreatic adenocarcinoma. Cancer Biol Ther 2008; 7: 1146–1156. 18535405 10.4161/cbt.7.7.6208 PMC2763640

[26] Lehmann U, Hasemeier B, Christgen M, Muller M, Romermann D, Länger F, et al Epigenetic inactivation of microRNA gene hsa-mir-9-1 in human breast cancer. J Pathol 2008; 214: 17–24. 10.1002/path.2251 17948228

[27] Peters I, Dubrowinskaja N, Abbas M, Seidel C, Kogosov M, Scherer R, et al DNA methylation biomarkers predict progression-free and overall survival of metastatic renal cell cancer (mRCC) treated with antiangiogenic therapies. PLoS One 2014; 9: e91440 10.1371/journal.pone.0091440 24633192 PMC3954691

[28] Anwar SL, Albat C, Krech T, Hasemeier B, Schipper E, Schweitzer N, et al Concordant hypermethylation of intergenic microRNA genes in human hepatocellular carcinoma as new diagnostic and prognostic marker. Int J Cancer 2013; 133: 660–670. 10.1002/ijc.28068 23364900

[29] Hong CC, Chen PS, Chiou J, Chiu CF, Yang CY, Hsiao M, et al miR326 maturation is crucial for VEGF-C-driven cortactin expression and esophageal cancer progression. Cancer Res 2014; 74: 6280–6290. 10.1158/0008-5472.CAN-14-0524 25205106

[30] Wang S, Lu S, Geng S, Ma S, Liang Z, Jiao B. Expression and clinical significance of microRNA-326 in human glioma miR-326 expression in glioma. Med Oncol 2013; 30: 373 10.1007/s12032-012-0373-y 23292865

[31] Cao G, Dong W, Meng X, Liu H, Liao H, Liu S. MiR-511 inhibits growth and metastasis of human hepatocellular carcinoma cells by targeting PIK3R3. Tumour Biol. 2015; (In press). 10.1007/s13277-015-3085-z 25608840

[32] Yao J, Liang L, Huang S, Ding J, Tan N, Zhao Y, et al MicroRNA-30d promotes tumor invasion and metastasis by targeting Galphai2 in hepatocellular carcinoma. Hepatology 2010; 51: 846–856. 10.1002/hep.23443 20054866

[33] Yu H, Lin X, Wang F, Zhang B, Wang W, Shi H, et al Proliferation inhibition and the underlying molecular mechanisms of microRNA-30d in renal carcinoma cells. Oncol Lett 2014; 7: 799–804. 10.3892/ol.2013.1754 24520297 PMC3919943

[34] Lee H, Park CS, Deftereos G, Morihara J, Stern JE, Hawes SE, et al MicroRNA expression in ovarian carcinoma and its correlation with clinicopathological features. World J Surg Oncol 2012; 10: 174 10.1186/1477-7819-10-174 22925189 PMC3449188

[35] Zhang P, Garnett J, Creighton CJ, Al Sannaa GA, Igram DR, Lazar A, et al (2014) EZH2-miR-30d-KPNB1 pathway regulates malignant peripheral nerve sheath tumour cell survival and tumourigenesis. J Pathol 232: 308–318. 10.1002/path.4294 24132643 PMC4166508

[36] Yang X, Zhong X, Tanyi JL, Shen J, Xu C, Gao P, et al mir-30d Regulates multiple genes in the autophagy pathway and impairs autophagy process in human cancer cells. Biochem Biophys Res Commun 2013; 431: 617–622. 10.1016/j.bbrc.2012.12.083 23274497 PMC3578012

[37] Wu C, Jin B, Chen L, Zhuo D, Zhang Z, Gong K, et al MiR-30d induces apoptosis and is regulated by the Akt/FOXO pathway in renal cell carcinoma. Cell Signal 2013; 25: 1212–1221. 10.1016/j.cellsig.2013.01.028 23416459

[38] LaCroix B, Gamazon ER, Lenkala D, Im HK, Geeleher P, Ziliak D, et al Integrative analyses of genetic variation, epigenetic regulation, and the transcriptome to elucidate the biology of platinum sensitivity. BMC Genomics 2014; 15: 292 10.1186/1471-2164-15-292 24739237 PMC3996490

[39] Zhuang LP, Meng ZQ. Serum miR-224 Reflects Stage of Hepatocellular Carcinoma and Predicts Survival. Biomed Res Int 2015; 2015: 731781 10.1155/2015/731781 25688365 PMC4320918

