# Supplementary material for: A Seven-microRNA Expression Signature Predicts Survival in Hepatocellular Carcinoma
Source: PLoS One. 2015 Jun 5;10(6):e0128628. doi: 10.1371/journal.pone.0128628 (PMC4457814; doi:10.1371/journal.pone.0128628)
Supplement: S1 Table — (DOCX) [file pone.0128628.s001.docx]

**S1 Table. Summary of miRNAs expressed differentially between tumor and adjacent non-tumor tissues**

| **MicroRNA** | **Expression Level ^a^** | | **Fold Change ^b^** | **P value** | **FDR** |
| --- | --- | --- | --- | --- | --- |
|  | Normal | Tumor |  |  |  |
| hsa-mir-1269 | 4.86 | 64.74 | 13.32 | 0.0000001 | 0.000000674 |
| hsa-mir-10b | 1606.74 | 11657.67 | 7.26 | < 1e-07 | < 1e-07 |
| hsa-mir-1251 | 0.21 | 1.31 | 6.24 | 0.0004089 | 0.00134 |
| hsa-mir-224 | 18.94 | 106.45 | 5.62 | < 1e-07 | < 1e-07 |
| hsa-mir-96 | 1.07 | 5.86 | 5.48 | < 1e-07 | < 1e-07 |
| hsa-mir-135a-1 | 0.46 | 2.25 | 4.89 | < 1e-07 | < 1e-07 |
| hsa-mir-183 | 286.31 | 1390.89 | 4.86 | < 1e-07 | < 1e-07 |
| hsa-mir-891a | 0.41 | 1.69 | 4.12 | 0.0003402 | 0.00112 |
| hsa-mir-182 | 1085.56 | 4016.83 | 3.70 | 0.0000003 | 0.00000194 |
| hsa-mir-184 | 0.57 | 2.05 | 3.60 | 0.0003469 | 0.00114 |
| hsa-mir-452 | 53.18 | 188.21 | 3.54 | < 1e-07 | < 1e-07 |
| hsa-mir-1266 | 1.62 | 5.09 | 3.14 | < 1e-07 | < 1e-07 |
| hsa-mir-34c | 0.52 | 1.56 | 3.00 | 0.0000005 | 0.00000308 |
| hsa-mir-190b | 0.27 | 0.77 | 2.85 | 0.0000064 | 0.0000342 |
| hsa-mir-3662 | 0.27 | 0.74 | 2.74 | 0.0000555 | 0.000227 |
| hsa-mir-93 | 2106.42 | 5760.2 | 2.73 | < 1e-07 | < 1e-07 |
| hsa-mir-508 | 1.14 | 3.11 | 2.73 | 0.0000137 | 0.0000677 |
| hsa-mir-3200 | 0.82 | 2.23 | 2.72 | 0.0000011 | 0.00000659 |
| hsa-mir-21 | 80519.31 | 216940.45 | 2.69 | < 1e-07 | < 1e-07 |
| hsa-mir-514-2 | 0.36 | 0.9 | 2.50 | 0.0001994 | 0.000709 |
| hsa-mir-1270-1 | 0.33 | 0.82 | 2.48 | 0.0000294 | 0.000129 |
| hsa-mir-9-1 | 56.11 | 139.03 | 2.48 | 0.000223 | 0.000776 |
| hsa-mir-1295 | 0.51 | 1.25 | 2.45 | 0.0001836 | 0.000656 |
| hsa-mir-9-2 | 56.68 | 138.89 | 2.45 | 0.0002636 | 0.000894 |
| hsa-mir-877 | 0.69 | 1.63 | 2.36 | < 1e-07 | < 1e-07 |
| hsa-mir-2114 | 0.51 | 1.2 | 2.35 | 0.0004316 | 0.0014 |
| hsa-mir-500a | 155.5 | 362.55 | 2.33 | < 1e-07 | < 1e-07 |
| hsa-mir-1270-2 | 0.35 | 0.81 | 2.31 | 0.000019 | 0.0000917 |
| hsa-mir-589 | 42.74 | 97.96 | 2.29 | < 1e-07 | < 1e-07 |
| hsa-mir-1180 | 9.13 | 20.48 | 2.24 | < 1e-07 | < 1e-07 |
| hsa-mir-221 | 54.2 | 113.32 | 2.09 | < 1e-07 | < 1e-07 |
| hsa-mir-1254 | 0.24 | 0.5 | 2.08 | 0.0001673 | 0.000612 |
| hsa-mir-301b | 0.56 | 1.16 | 2.07 | 0.0000804 | 0.000319 |
| hsa-mir-3677 | 1.86 | 3.83 | 2.06 | 0.0000011 | 0.00000659 |
| hsa-mir-760 | 0.35 | 0.72 | 2.06 | 0.0000261 | 0.000116 |
| hsa-mir-532 | 709.2 | 1445.6 | 2.04 | < 1e-07 | < 1e-07 |
| hsa-mir-501 | 30.03 | 60.08 | 2.00 | < 1e-07 | < 1e-07 |
| hsa-mir-937 | 0.94 | 1.88 | 2.00 | 0.0000239 | 0.000109 |
| hsa-mir-660 | 40.27 | 80.41 | 2.00 | < 1e-07 | < 1e-07 |
| hsa-mir-19a | 22.67 | 45.02 | 1.99 | 0.000002 | 0.0000117 |
| hsa-mir-4326 | 2.42 | 4.8 | 1.98 | 0.0000192 | 0.000092 |
| hsa-mir-1226 | 0.53 | 1.05 | 1.98 | 0.0000448 | 0.000187 |
| hsa-mir-34a | 164.11 | 314.93 | 1.92 | < 1e-07 | < 1e-07 |
| hsa-mir-222 | 15.42 | 28.86 | 1.87 | 0.0000001 | 0.000000674 |
| hsa-mir-20a | 323.91 | 604.47 | 1.87 | 0.0000006 | 0.00000366 |
| hsa-mir-500b | 3.86 | 7.09 | 1.84 | < 1e-07 | < 1e-07 |
| hsa-mir-18a | 9.03 | 16.57 | 1.83 | 0.0001437 | 0.000539 |
| hsa-mir-939 | 0.47 | 0.86 | 1.83 | 0.0000036 | 0.0000208 |
| hsa-mir-581 | 0.38 | 0.69 | 1.82 | 0.0000049 | 0.0000269 |
| hsa-mir-643 | 0.46 | 0.82 | 1.78 | 0.0000315 | 0.000136 |
| hsa-mir-1301 | 5.24 | 9.31 | 1.78 | 0.0000067 | 0.0000352 |
| hsa-mir-1229 | 0.35 | 0.62 | 1.77 | 0.0002389 | 0.000827 |
| hsa-mir-3922 | 0.34 | 0.6 | 1.76 | 0.0001289 | 0.000494 |
| hsa-mir-103-1 | 15513.63 | 27220.56 | 1.75 | < 1e-07 | < 1e-07 |
| hsa-mir-3682 | 0.77 | 1.35 | 1.75 | 0.0000428 | 0.00018 |
| hsa-mir-421 | 2.52 | 4.36 | 1.73 | 0.0000046 | 0.0000257 |
| hsa-mir-30d | 7814.98 | 13285.85 | 1.70 | < 1e-07 | < 1e-07 |
| hsa-mir-1292 | 0.3 | 0.51 | 1.70 | 0.0002461 | 0.000842 |
| hsa-mir-362 | 15.04 | 25.39 | 1.69 | 0.0000042 | 0.0000238 |
| hsa-mir-3127 | 1.62 | 2.73 | 1.69 | 0.0000202 | 0.0000954 |
| hsa-mir-3610 | 0.35 | 0.58 | 1.66 | 0.0006113 | 0.00196 |
| hsa-mir-301a | 4.63 | 7.61 | 1.64 | 0.0001255 | 0.000486 |
| hsa-mir-502 | 8.62 | 13.89 | 1.61 | < 1e-07 | 0.000000674 |
| hsa-mir-17 | 771.79 | 1228.89 | 1.59 | 0.0000249 | 0.000112 |
| hsa-mir-454 | 4.61 | 7.3 | 1.58 | 0.0000071 | 0.000037 |
| hsa-mir-25 | 6103.15 | 9622.98 | 1.58 | < 1e-07 | < 1e-07 |
| hsa-mir-92a-1 | 784.35 | 1229.98 | 1.57 | 0.000103 | 0.000404 |
| hsa-mir-19b-2 | 126.55 | 197.03 | 1.56 | 0.0002045 | 0.000723 |
| hsa-mir-1307 | 1374.09 | 2042.39 | 1.49 | 0.0000057 | 0.0000307 |
| hsa-mir-103-2 | 7.24 | 10.74 | 1.48 | 0.0000895 | 0.000353 |
| hsa-mir-425 | 119.49 | 176.4 | 1.48 | 0.000129 | 0.000494 |
| hsa-mir-140 | 1172.78 | 1656.41 | 1.41 | < 1e-07 | < 1e-07 |
| hsa-mir-106b | 362.02 | 507.13 | 1.40 | 0.000028 | 0.000123 |
| hsa-mir-423 | 126.9 | 173.12 | 1.36 | 0.0000005 | 0.00000308 |
| hsa-mir-185 | 85.84 | 116.59 | 1.36 | 0.0000208 | 0.0000975 |
| hsa-mir-769 | 14.72 | 19.03 | 1.29 | 0.0002412 | 0.00083 |
| hsa-mir-15a | 166.47 | 214.86 | 1.29 | 0.0009171 | 0.00289 |
| hsa-let-7a-1 | 9103.47 | 7002.48 | 0.77 | 0.0001782 | 0.000641 |
| hsa-let-7a-3 | 9132.53 | 7020.07 | 0.77 | 0.0001761 | 0.000638 |
| hsa-mir-191 | 707.26 | 543.08 | 0.77 | 0.0009889 | 0.00309 |
| hsa-let-7a-2 | 18213.63 | 13963.78 | 0.77 | 0.0001535 | 0.000569 |
| hsa-mir-193a | 726.27 | 538.61 | 0.74 | 0.0001765 | 0.000638 |
| hsa-mir-16-1 | 591.4 | 428.55 | 0.72 | 0.000014 | 0.0000686 |
| hsa-mir-23b | 1979.06 | 1411.69 | 0.71 | 0.00002 | 0.0000951 |
| hsa-mir-30b | 829.64 | 590.7 | 0.71 | 0.0002232 | 0.000776 |
| hsa-mir-744 | 48.46 | 34.29 | 0.71 | 0.0001441 | 0.000539 |
| hsa-mir-30c-2 | 950.21 | 669.06 | 0.70 | 0.0000232 | 0.000106 |
| hsa-mir-598 | 17.49 | 12.17 | 0.70 | 0.0006384 | 0.00203 |
| hsa-let-7g | 1069 | 737.39 | 0.69 | 0.0000044 | 0.0000248 |
| hsa-mir-7-1 | 19.14 | 13.2 | 0.69 | 0.0001594 | 0.000586 |
| hsa-mir-328 | 44.17 | 30.46 | 0.69 | 0.0000268 | 0.000119 |
| hsa-mir-22 | 194033.06 | 127066.75 | 0.65 | < 1e-07 | < 1e-07 |
| hsa-mir-624 | 4.74 | 3.02 | 0.64 | 0.0000229 | 0.000106 |
| hsa-mir-126 | 7617.83 | 4809.31 | 0.63 | < 1e-07 | < 1e-07 |
| hsa-mir-663 | 0.69 | 0.43 | 0.62 | 0.0009686 | 0.00304 |
| hsa-mir-345 | 25.38 | 15.8 | 0.62 | 0.0000351 | 0.000148 |
| hsa-mir-3199-2 | 1.08 | 0.67 | 0.62 | 0.0000223 | 0.000104 |
| hsa-mir-574 | 192.81 | 118.83 | 0.62 | < 1e-07 | < 1e-07 |
| hsa-mir-148a | 140515.63 | 86321.38 | 0.61 | 0.0000048 | 0.0000265 |
| hsa-mir-143 | 57538.24 | 34906.68 | 0.61 | 0.0001382 | 0.000523 |
| hsa-mir-33a | 54.15 | 32.64 | 0.60 | 0.0000349 | 0.000148 |
| hsa-mir-3157 | 0.86 | 0.51 | 0.59 | 0.0000123 | 0.0000612 |
| hsa-mir-27b | 3510.22 | 2032.09 | 0.58 | < 1e-07 | < 1e-07 |
| hsa-mir-146a | 98.77 | 56.64 | 0.57 | 0.0008908 | 0.00283 |
| hsa-mir-628 | 17.72 | 10.1 | 0.57 | < 1e-07 | < 1e-07 |
| hsa-mir-100 | 7846.32 | 4464.99 | 0.57 | 0.0005874 | 0.00189 |
| hsa-mir-665 | 0.76 | 0.43 | 0.57 | 0.0005264 | 0.0017 |
| hsa-mir-30c-1 | 4.17 | 2.33 | 0.56 | < 1e-07 | < 1e-07 |
| hsa-mir-30a | 31677.49 | 17332.62 | 0.55 | 0.0000004 | 0.00000254 |
| hsa-mir-1248 | 1.81 | 0.98 | 0.54 | 0.0000747 | 0.000302 |
| hsa-mir-30e | 29017.5 | 15669.56 | 0.54 | < 1e-07 | < 1e-07 |
| hsa-mir-29a | 11803.24 | 6283.73 | 0.53 | < 1e-07 | < 1e-07 |
| hsa-mir-551a | 0.55 | 0.29 | 0.53 | 0.0000111 | 0.0000561 |
| hsa-mir-133b | 0.84 | 0.44 | 0.52 | 0.0000066 | 0.000035 |
| hsa-mir-3687 | 1.34 | 0.7 | 0.52 | 0.0000787 | 0.000314 |
| hsa-mir-125a | 506.64 | 258.59 | 0.51 | 0.0000002 | 0.00000131 |
| hsa-mir-26a-1 | 2.53 | 1.29 | 0.51 | < 1e-07 | < 1e-07 |
| hsa-mir-101-2 | 165.9 | 84.5 | 0.51 | < 1e-07 | < 1e-07 |
| hsa-mir-152 | 535.47 | 272.34 | 0.51 | < 1e-07 | < 1e-07 |
| hsa-let-7b | 12039.64 | 6083.98 | 0.51 | < 1e-07 | < 1e-07 |
| hsa-mir-1271 | 2.72 | 1.37 | 0.50 | < 1e-07 | 0.000000674 |
| hsa-mir-215 | 190.46 | 94.93 | 0.50 | 0.0000785 | 0.000314 |
| hsa-mir-26a-2 | 4000.34 | 1987.74 | 0.50 | < 1e-07 | < 1e-07 |
| hsa-mir-627 | 2.63 | 1.28 | 0.49 | < 1e-07 | < 1e-07 |
| hsa-mir-370 | 12.15 | 5.9 | 0.49 | 0.0002861 | 0.000959 |
| hsa-mir-223 | 232.72 | 112.41 | 0.48 | 0.0000004 | 0.00000254 |
| hsa-mir-376b | 3.26 | 1.55 | 0.48 | 0.0002112 | 0.000743 |
| hsa-mir-874 | 37.8 | 17.8 | 0.47 | 0.0000012 | 0.00000712 |
| hsa-mir-505 | 136.27 | 64.12 | 0.47 | < 1e-07 | < 1e-07 |
| hsa-mir-494 | 2.42 | 1.13 | 0.47 | 0.0003235 | 0.00107 |
| hsa-mir-3065 | 55.8 | 25.72 | 0.46 | 0.0000316 | 0.000136 |
| hsa-mir-26b | 2205.8 | 1012.32 | 0.46 | < 1e-07 | < 1e-07 |
| hsa-mir-1468 | 23.83 | 10.71 | 0.45 | 0.0000001 | 0.000000674 |
| hsa-mir-122 | 37794.86 | 16946.72 | 0.45 | 0.0000346 | 0.000148 |
| hsa-mir-127 | 850.76 | 380.36 | 0.45 | 0.0000245 | 0.000111 |
| hsa-mir-381 | 67.27 | 29.81 | 0.44 | 0.0000099 | 0.0000504 |
| hsa-mir-3676 | 2.74 | 1.21 | 0.44 | 0.0000013 | 0.00000765 |
| hsa-mir-378 | 1735.38 | 759.32 | 0.44 | < 1e-07 | < 1e-07 |
| hsa-mir-376a-1 | 2.72 | 1.19 | 0.44 | 0.0001357 | 0.000516 |
| hsa-mir-621 | 0.88 | 0.38 | 0.43 | < 1e-07 | < 1e-07 |
| hsa-mir-154 | 8.97 | 3.84 | 0.43 | 0.000154 | 0.000569 |
| hsa-mir-377 | 4.63 | 1.96 | 0.42 | 0.0000481 | 0.000198 |
| hsa-mir-455 | 1571.21 | 662.31 | 0.42 | < 1e-07 | < 1e-07 |
| hsa-mir-153-2 | 12.07 | 4.97 | 0.41 | < 1e-07 | < 1e-07 |
| hsa-mir-299 | 5.89 | 2.4 | 0.41 | 0.0000123 | 0.0000612 |
| hsa-mir-150 | 613.42 | 249.14 | 0.41 | 0.0000005 | 0.00000308 |
| hsa-mir-203 | 1103.24 | 441.45 | 0.40 | 0.0003238 | 0.00107 |
| hsa-mir-136 | 51.92 | 20.71 | 0.40 | 0.0000099 | 0.0000504 |
| hsa-mir-125b-1 | 1134.63 | 448.62 | 0.40 | < 1e-07 | < 1e-07 |
| hsa-mir-24-1 | 75.9 | 29.99 | 0.40 | < 1e-07 | < 1e-07 |
| hsa-mir-200b | 107.66 | 41.23 | 0.38 | 0.0002639 | 0.000894 |
| hsa-mir-200a | 116.08 | 44.29 | 0.38 | 0.0002782 | 0.000937 |
| hsa-mir-542 | 517.4 | 196.91 | 0.38 | < 1e-07 | < 1e-07 |
| hsa-mir-497 | 35.68 | 13.55 | 0.38 | < 1e-07 | < 1e-07 |
| hsa-mir-323 | 5.29 | 1.99 | 0.38 | 0.0001052 | 0.00041 |
| hsa-mir-450b | 26.76 | 9.95 | 0.37 | < 1e-07 | < 1e-07 |
| hsa-mir-101-1 | 44546.81 | 16485.36 | 0.37 | < 1e-07 | < 1e-07 |
| hsa-mir-134 | 346.61 | 127.74 | 0.37 | 0.0000039 | 0.0000223 |
| hsa-mir-3130-1 | 6.85 | 2.51 | 0.37 | 0.0000002 | 0.00000131 |
| hsa-mir-99a | 1572.21 | 572.46 | 0.36 | < 1e-07 | < 1e-07 |
| hsa-mir-3653 | 16.45 | 5.98 | 0.36 | < 1e-07 | < 1e-07 |
| hsa-mir-378c | 36.31 | 13.1 | 0.36 | < 1e-07 | < 1e-07 |
| hsa-mir-29c | 4302.17 | 1545.29 | 0.36 | < 1e-07 | < 1e-07 |
| hsa-mir-3647 | 11.9 | 4.27 | 0.36 | < 1e-07 | < 1e-07 |
| hsa-mir-429 | 19.55 | 6.79 | 0.35 | 0.0000619 | 0.000252 |
| hsa-mir-592 | 5.24 | 1.81 | 0.35 | 0.0000456 | 0.000189 |
| hsa-mir-142 | 3142.81 | 1068.46 | 0.34 | < 1e-07 | < 1e-07 |
| hsa-mir-145 | 2221.9 | 751.07 | 0.34 | < 1e-07 | < 1e-07 |
| hsa-mir-3614 | 8.09 | 2.72 | 0.34 | < 1e-07 | < 1e-07 |
| hsa-mir-369 | 23 | 7.73 | 0.34 | 0.0000002 | 0.00000131 |
| hsa-mir-125b-2 | 57.73 | 19.28 | 0.33 | < 1e-07 | < 1e-07 |
| hsa-mir-187 | 3.7 | 1.22 | 0.33 | 0.0000097 | 0.0000502 |
| hsa-mir-130a | 131.37 | 43.3 | 0.33 | < 1e-07 | < 1e-07 |
| hsa-mir-10a | 29145.03 | 9564.71 | 0.33 | < 1e-07 | < 1e-07 |
| hsa-mir-337 | 54.06 | 17.44 | 0.32 | < 1e-07 | < 1e-07 |
| hsa-let-7c | 5484.65 | 1681.38 | 0.31 | < 1e-07 | < 1e-07 |
| hsa-mir-654 | 54.16 | 16.52 | 0.31 | < 1e-07 | < 1e-07 |
| hsa-mir-758 | 33.81 | 10.22 | 0.30 | < 1e-07 | 0.000000674 |
| hsa-mir-379 | 1294.49 | 390.46 | 0.30 | < 1e-07 | < 1e-07 |
| hsa-mir-450a-2 | 10.69 | 3.2 | 0.30 | < 1e-07 | < 1e-07 |
| hsa-mir-511-2 | 16.5 | 4.84 | 0.29 | < 1e-07 | < 1e-07 |
| hsa-mir-511-1 | 16.84 | 4.92 | 0.29 | < 1e-07 | < 1e-07 |
| hsa-mir-144 | 343.4 | 100.29 | 0.29 | < 1e-07 | < 1e-07 |
| hsa-mir-450a-1 | 10.86 | 3.17 | 0.29 | < 1e-07 | < 1e-07 |
| hsa-mir-486 | 292.18 | 83.84 | 0.29 | < 1e-07 | < 1e-07 |
| hsa-mir-326 | 11.45 | 3.14 | 0.27 | < 1e-07 | < 1e-07 |
| hsa-mir-195 | 87.29 | 23.49 | 0.27 | < 1e-07 | < 1e-07 |
| hsa-mir-375 | 5288.31 | 1377.52 | 0.26 | 0.0000183 | 0.000089 |
| hsa-mir-376c | 13.63 | 3.53 | 0.26 | < 1e-07 | < 1e-07 |
| hsa-mir-411 | 19.99 | 4.6 | 0.23 | < 1e-07 | < 1e-07 |
| hsa-mir-451 | 1509.89 | 339.7 | 0.22 | < 1e-07 | < 1e-07 |
| hsa-mir-383 | 6.51 | 1.45 | 0.22 | 0.0000001 | 0.000000674 |
| hsa-mir-139 | 458.42 | 100.98 | 0.22 | < 1e-07 | < 1e-07 |
| hsa-mir-1274b | 7.91 | 1.72 | 0.22 | < 1e-07 | < 1e-07 |
| hsa-mir-3607 | 192.84 | 41.42 | 0.21 | < 1e-07 | < 1e-07 |
| hsa-mir-214 | 33.5 | 7.06 | 0.21 | < 1e-07 | < 1e-07 |
| hsa-mir-3648 | 8.86 | 1.82 | 0.21 | < 1e-07 | < 1e-07 |
| hsa-mir-33b | 22.76 | 4.66 | 0.20 | < 1e-07 | < 1e-07 |
| hsa-mir-199a-1 | 1425.71 | 287.93 | 0.20 | < 1e-07 | < 1e-07 |
| hsa-mir-199a-2 | 2298.19 | 448.22 | 0.20 | < 1e-07 | < 1e-07 |
| hsa-mir-199b | 2584.4 | 477.18 | 0.18 | < 1e-07 | < 1e-07 |
| hsa-mir-1247 | 29.6 | 4.98 | 0.17 | < 1e-07 | < 1e-07 |
| hsa-mir-675 | 323.45 | 51.72 | 0.16 | < 1e-07 | < 1e-07 |
| hsa-mir-424 | 615.1 | 90.84 | 0.15 | < 1e-07 | < 1e-07 |
| hsa-mir-483 | 105.97 | 14.48 | 0.14 | 0.0000052 | 0.0000283 |
| hsa-mir-490 | 7.68 | 0.81 | 0.11 | < 1e-07 | < 1e-07 |
| hsa-mir-1258 | 13.08 | 0.92 | 0.07 | < 1e-07 | < 1e-07 |
| FDR, false discovery rate a log2 transformed expression data b geometric mean of the ratio between tumor and matched non-tumor miRNAs (expressed as reads per million) | | | | | |
