# Supplementary material for: A Seven-microRNA Expression Signature Predicts Survival in Hepatocellular Carcinoma
Source: PLoS One. 2015 Jun 5;10(6):e0128628. doi: 10.1371/journal.pone.0128628 (PMC4457814; doi:10.1371/journal.pone.0128628)
Supplement: S2 Table — (DOCX) [file pone.0128628.s002.docx]

**S2 Table 1-9. Differentially expressed miRNAs from Tumor/Non-tumor according to clinical parameters.**

**S2 Table 1. Summary of miRNAs expressed differentially between different genders (Female vs. Male).**

| **MicroRNA** | **Expression Level** ^a^ | | **Fold Change** ^b^ | **P** value | **FDR** |
| --- | --- | --- | --- | --- | --- |
|  | **Male** | **Female** |  |  |  |
| hsa-mir-1468 | 12.02 | 8.19 | 0.68 | 0.0008581 | 0.0127 |
| hsa-let-7c | 1885.89 | 1306.96 | 0.69 | 0.0002352 | 0.0051 |
| hsa-mir-125b-2 | 21.64 | 15.02 | 0.69 | 0.000258 | 0.0051 |
| hsa-mir-140 | 1739.93 | 1487.18 | 0.85 | 0.0002007 | 0.0051 |
| hsa-mir-26a-2 | 1865.12 | 2292.86 | 1.23 | 0.000059 | 0.00407 |
| hsa-mir-26b | 943.71 | 1180.99 | 1.25 | 0.000221 | 0.0051 |
| hsa-mir-1301 | 8.34 | 11.97 | 1.44 | 0.0001523 | 0.0051 |
| hsa-mir-1266 | 4.44 | 6.96 | 1.57 | 0.0003006 | 0.00519 |
| hsa-mir-3065 | 21.33 | 38.44 | 1.80 | 0.000029 | 0.003 |
| hsa-mir-182 | 3201.56 | 6485.58 | 2.03 | 0.0002653 | 0.0051 |
| hsa-mir-96 | 4.71 | 9.54 | 2.03 | 0.0002709 | 0.0051 |
| hsa-mir-183 | 1088.8 | 2340.97 | 2.15 | 0.0002544 | 0.0051 |
| hsa-mir-1247 | 3.77 | 9.22 | 2.45 | 0.0003959 | 0.0063 |
| hsa-mir-375 | 996.03 | 2854.56 | 2.87 | 0.0000092 | 0.0019 |
| hsa-mir-483 | 10.22 | 31.04 | 3.04 | 0.0009319 | 0.0129 |

FDR, false discovery rate
a log2 transformed expression data
b geometric mean of the ratio between different genders (Female vs. Male)

**S2 Table 2. Summary of miRNAs expressed differentially between different races (White vs. Asian).**

| **MicroRNA** | **Expression Level** ^a^ | | **Fold Change** ^b^ | **P** value | **FDR** |
| --- | --- | --- | --- | --- | --- |
|  | **Asian** | **White** |  |  |  |
| hsa-mir-4326 | 5.95 | 3.99 | 0.67 | 0.0002466 | 0.00567 |
| hsa-mir-1180 | 25.25 | 17.49 | 0.69 | 0.0002985 | 0.00618 |
| hsa-mir-30d | 15864.24 | 11237.9 | 0.71 | 0.0000008 | 0.0000828 |
| hsa-mir-532 | 1710 | 1242.03 | 0.73 | 0.0000017 | 0.000117 |
| hsa-mir-17 | 1415.61 | 1076.95 | 0.76 | 0.0006247 | 0.00995 |
| hsa-mir-26a-2 | 1787.73 | 2173.83 | 1.22 | 0.0000635 | 0.00219 |
| hsa-mir-30e | 14071.55 | 17167.14 | 1.22 | 0.0000899 | 0.00266 |
| hsa-mir-29a | 5549.68 | 6893.47 | 1.24 | 0.000904 | 0.0125 |
| hsa-mir-26b | 888.05 | 1144.17 | 1.29 | 0.0000134 | 0.000693 |
| hsa-mir-511-2 | 4.02 | 5.51 | 1.37 | 0.0004575 | 0.00861 |
| hsa-mir-511-1 | 4.07 | 5.58 | 1.37 | 0.0006028 | 0.00995 |
| hsa-mir-627 | 1.08 | 1.56 | 1.44 | 0.0000248 | 0.00103 |
| hsa-mir-10b | 8934.21 | 14594 | 1.63 | 0.0008805 | 0.0125 |
| hsa-mir-3130-1 | 1.49 | 3.82 | 2.56 | < 1e-07 | < 1e-07 |
| hsa-mir-1269 | 35.54 | 126.8 | 3.57 | 0.0001819 | 0.00471 |

FDR, false discovery rate
a log2 transformed expression data
b geometric mean of the ratio between different races (White vs. Asian)

**S2 Table 3. Summary of miRNAs expressed differentially between different risk factors (No vs. Yes).**

| **MicroRNA** | **Expression Level** ^a^ | | **Fold Change** ^b^ | **P** value | **FDR** |
| --- | --- | --- | --- | --- | --- |
|  | **Yes** | **No** |  |  |  |
| hsa-mir-3676 | 1.06 | 1.77 | 1.67 | 0.0003643 | 0.0754 |

FDR, false discovery rate
a log2 transformed expression data
b geometric mean of the ratio different risk factors (No vs. Yes)

**S2 Table 4. Summary of miRNAs expressed differentially between different tumor grades (G3+G4 vs. G1+G2).**

| **MicroRNA** | **Expression Level** ^a^ | | **Fold Change** ^b^ | **P** value | **FDR** |
| --- | --- | --- | --- | --- | --- |
|  | **G1+G2** | **G3+G4** |  |  |  |
| hsa-mir-100 | 5358.34 | 3322.87 | 0.62 | 0.0000639 | 0.00331 |
| hsa-mir-378c | 15.34 | 10.39 | 0.68 | 0.000137 | 0.00567 |
| hsa-mir-99a | 663.47 | 459.57 | 0.69 | 0.0003813 | 0.00789 |
| hsa-mir-125b-1 | 513.99 | 361.07 | 0.70 | 0.000005 | 0.00104 |
| hsa-mir-378 | 873.21 | 619.62 | 0.71 | 0.0005463 | 0.00808 |
| hsa-mir-505 | 71.73 | 53.57 | 0.75 | 0.0005145 | 0.00808 |
| hsa-mir-101-1 | 18198.54 | 14094.04 | 0.77 | 0.0000547 | 0.00331 |
| hsa-mir-29c | 1715.82 | 1328.45 | 0.77 | 0.0009469 | 0.0123 |
| hsa-mir-22 | 137958.67 | 112632.24 | 0.82 | 0.0002353 | 0.00732 |
| hsa-mir-423 | 162.63 | 190.99 | 1.17 | 0.0002828 | 0.00732 |
| hsa-mir-93 | 5267.42 | 6674.06 | 1.27 | 0.0003784 | 0.00789 |
| hsa-mir-3677 | 3.33 | 4.82 | 1.45 | 0.0004356 | 0.00808 |
| hsa-mir-4326 | 3.98 | 6.42 | 1.61 | 0.0000137 | 0.00142 |
| hsa-mir-1270-1 | 0.69 | 1.1 | 1.59 | 0.0008712 | 0.012 |
| hsa-mir-1270-2 | 0.67 | 1.11 | 1.66 | 0.0002485 | 0.00732 |
| hsa-mir-3200 | 1.87 | 3.09 | 1.65 | 0.0004854 | 0.00808 |

FDR, false discovery rate
a log2 transformed expression data
b geometric mean of the ratio between tumor grades (G3+G4 vs. G1+G2)

**S2 Table 5. Summary of miRNAs expressed differentially between different AJCC TNM staging system T (T3+T4 vs. T1+T2).**

| **MicroRNA** | **Expression Level** ^a^ | | **Fold Change** ^b^ | **P** value | **FDR** |
| --- | --- | --- | --- | --- | --- |
|  | **T1+T2** | **T3+T4** |  |  |  |
| hsa-mir-122 | 20402.21 | 9862.42 | 0.48 | 0.0000031 | 0.000642 |
| hsa-mir-148a | 93001.51 | 68188.05 | 0.73 | 0.0002768 | 0.00846 |
| hsa-mir-22 | 134706.89 | 107547.85 | 0.80 | 0.0002479 | 0.00846 |
| hsa-mir-25 | 9112.47 | 11260.17 | 1.24 | 0.0003729 | 0.00965 |
| hsa-mir-454 | 6.75 | 9.1 | 1.35 | 0.000286 | 0.00846 |
| hsa-mir-3127 | 2.5 | 3.6 | 1.44 | 0.0001903 | 0.00846 |
| hsa-mir-326 | 2.78 | 4.5 | 1.62 | 0.0000141 | 0.00146 |
| hsa-mir-133b | 0.39 | 0.68 | 1.74 | 0.0002186 | 0.00846 |

FDR, false discovery rate
a log2 transformed expression data
b geometric mean of the ratio between different AJCC TNM staging system T (T3+T4 vs. T1+T2)

**S2 Table 6. Summary of miRNAs expressed differentially between different AJCC TNM staging system N (N1 vs. N0).**

| **MicroRNA** | **Expression Level** ^a^ | | **Fold Change** ^b^ | **P** value | **FDR** |
| --- | --- | --- | --- | --- | --- |
|  | **N0** | **N1** |  |  |  |
| hsa-mir-125b-2 | 18.97 | 4.23 | 0.22 | 0.0005065 | 0.0524 |
| hsa-mir-891a | 1.57 | 252.37 | 160.75 | 0.0000078 | 0.00161 |

FDR, false discovery rate
a log2 transformed expression data
b geometric mean of the ratio between different AJCC TNM staging system N (N1 vs. N0)

**S2 Table 7. Summary of miRNAs expressed differentially between different AJCC pathological stage (III + IV vs. I + II).**

| **MicroRNA** | **Expression Level** ^a^ | | **Fold Change** ^b^ | **P** value | **FDR** |
| --- | --- | --- | --- | --- | --- |
|  | **I + II** | **III + IV** |  |  |  |
| hsa-mir-122 | 21423.08 | 10097.61 | 0.47 | 0.0000008 | 0.000166 |
| hsa-mir-148a | 94512.48 | 68194.81 | 0.72 | 0.0001577 | 0.00489 |
| hsa-mir-22 | 135286.65 | 104252.34 | 0.77 | 0.0000379 | 0.00262 |
| hsa-mir-25 | 9106.74 | 11343.61 | 1.25 | 0.0003308 | 0.00856 |
| hsa-mir-3127 | 2.48 | 3.55 | 1.43 | 0.0003863 | 0.00888 |
| hsa-mir-454 | 6.79 | 9.88 | 1.46 | 0.0000056 | 0.00058 |
| hsa-mir-1301 | 8.55 | 12.63 | 1.48 | 0.0001653 | 0.00489 |
| hsa-mir-301a | 6.99 | 10.68 | 1.53 | 0.0000664 | 0.00344 |
| hsa-mir-1248 | 0.88 | 1.35 | 1.53 | 0.0008641 | 0.0163 |
| hsa-mir-326 | 2.74 | 4.26 | 1.55 | 0.0001289 | 0.00489 |
| hsa-mir-133b | 0.39 | 0.66 | 1.69 | 0.0007442 | 0.0154 |

FDR, false discovery rate
a log2 transformed expression data
b geometric mean of the ratio between different AJCC pathological stage (III + IV vs. I + II)

**S2 Table 8. Summary of miRNAs expressed differentially between different new tumor events (Yes vs. No).**

| **MicroRNA** | **Expression Level** ^a^ | | **Fold Change** ^b^ | **P** value | **FDR** |
| --- | --- | --- | --- | --- | --- |
|  | **No** | **Yes** |  |  |  |
| hsa-mir-508 | 3.92 | 1.96 | 0.50 | 0.000093 | 0.0193 |
| hsa-mir-514-2 | 1.02 | 0.57 | 0.56 | 0.000831 | 0.086 |

FDR, false discovery rate
a log2 transformed expression data
b geometric mean of the ratio between different new tumor events (Yes vs. No)

**S2 Table 9. Summary of miRNAs expressed differentially between different Age at diagnosis (≥60 vs. <60).**

| **MicroRNA** | **Expression Level** ^a^ | | **Fold Change** ^b^ | **P** value | **FDR** |
| --- | --- | --- | --- | --- | --- |
|  | **<60** | **≥60** |  |  |  |
| hsa-mir-483 | 33.97 | 6.99 | 0.21 | 0.0000003 | 0.0000621 |
| hsa-mir-1180 | 25.53 | 17.16 | 0.67 | 0.0000377 | 0.0039 |
| hsa-mir-1301 | 11.09 | 8.08 | 0.73 | 0.0003507 | 0.0145 |
| hsa-mir-17 | 1445.88 | 1077.6 | 0.75 | 0.0001383 | 0.00954 |
| hsa-mir-29a | 5535.63 | 6993.06 | 1.26 | 0.000247 | 0.0128 |
| hsa-mir-891a | 1.15 | 2.31 | 2.01 | 0.0008916 | 0.0264 |
| hsa-mir-1269 | 34.51 | 109.1 | 3.16 | 0.0004336 | 0.015 |

FDR, false discovery rate
a log2 transformed expression data
b geometric mean of the ratio between different Age at diagnosis (≥60 vs. <60)
