# Supplementary material for: A Seven-microRNA Expression Signature Predicts Survival in Hepatocellular Carcinoma
Source: PLoS One. 2015 Jun 5;10(6):e0128628. doi: 10.1371/journal.pone.0128628 (PMC4457814; doi:10.1371/journal.pone.0128628)
Supplement: S3 Table — (DOCX) [file pone.0128628.s003.docx]

**S3 Table. miRNAs associated with overall survival by univariate Cox regression analysis.**

| **MicroRNA** | **Univariate test for miRNA** | | | **Cox proportional hazards models** |
| --- | --- | --- | --- | --- |
|  | **Hazard Ratio** | **P-value** | **FDR** | **P-value** |
| hsa-mir-30d | 0.632 | 0.00069 | 0.0215 | 0.000556 |
| hsa-mir-326 | 1.49 | 2.95E-05 | 0.00204 | 5.48E-05 |
| hsa-mir-3677 | 1.367 | 0.000728 | 0.0215 | 0.000787 |
| hsa-mir-511-1 | 1.49 | 0.000408 | 0.0169 | 0.0004 |
| hsa-mir-511-2 | 1.45 | 0.000888 | 0.023 | 0.000839 |
| hsa-mir-9-1 | 1.214 | 6.6E-06 | 0.00068 | 1.92E-05 |
| hsa-mir-9-2 | 1.215 | 6.2E-06 | 0.00068 | 1.81E-05 |

HR, hazard ratio; CI, confidential interval; FDR, false discovery rate.
