# Supplementary material for: A Seven-microRNA Expression Signature Predicts Survival in Hepatocellular Carcinoma
Source: PLoS One. 2015 Jun 5;10(6):e0128628. doi: 10.1371/journal.pone.0128628 (PMC4457814; doi:10.1371/journal.pone.0128628)
Supplement: S4 Table — (DOCX) [file pone.0128628.s004.docx]

**S4 Table. Cancer related and non-cancer related KEGG pathways enriched in seven miRNA target genes**

|  | **KEGG pathway** | **Number of miRNAs** | **Number of genes** | **P-value** | **List of genes** |
| --- | --- | --- | --- | --- | --- |
| Cancer related pathways | Prostate cancer | 5 | 30 | 0.000 | PDGFRA, NFKB1, CREB3L3, SOS2, PIK3CB, CREB5, TGFA, HSP90AA1, RAF1, CDKN1B, IGF1R, KRAS, AR, PIK3CD, PIK3R3, CCND1, CCNE2, PIK3R1, SOS1, EP300, AKT3, PDGFC, CREB3L2, PIK3CA, TCF7, PTEN, FGFR1, FOXO1, CREBBP, PDGFA |
|  | Melanoma | 4 | 21 | 0.000 | FGF12, PDGFRA, PIK3CB, FGF10, RAF1, FGF4, IGF1R, CDK6, MITF, CDH1, PIK3R3, CCND1, PIK3R1, FGF9, FGF2, FGF18, FGF5, PIK3CA, PTEN, FGFR1, FGF1 |
|  | Pathways in cancer | 2 | 38 | 0.001 | FGF12, FZD5, NFKB1, TCF4, PIK3CB, HSP90AA1, FGF10, RAF1, SMAD3, RARB, IKBKB, PTK2, CDH1, RUNX1T1, ARNT2, AR, PIK3R3, MSH6, CCNE2, RASSF5, DAPK1, ITGA2, SOS1, FGF9, FGF18, FLT3, FGF5, RALGDS, RALB, ITGA6, SHH, TCF7, PTEN, FOXO1, FGF1, TRAF3, TGFBR2, CCDC6 |
|  | Colorectal cancer | 2 | 10 | 0.003 | TCF4, RAF1, SMAD3, APPL1, MLH1, MSH6, PIK3R1, TGFB2, PIK3CA, TGFBR2 |
|  | Acute myeloid leukemia | 1 | 8 | 0.003 | NFKB1, PIK3CB, RAF1, RUNX1T1, PIK3R3, SOS1, FLT3, TCF7 |
|  | Renal cell carcinoma | 1 | 5 | 0.005 | TGFA, EPAS1, SOS1, AKT3, CREBBP |
|  | Glioma | 2 | 9 | 0.012 | PDGFRA, SOS2, TGFA, CDK6, PIK3R1, SOS1, AKT3, PIK3CA, PTEN |
|  | Chronic myeloid leukemia | 1 | 6 | 0.035 | SOS2, CDK6, PIK3R1, TGFB2, PIK3CA, TGFBR2 |
|  | Transcriptional misregulation in cancer | 4 | 45 | 0.000 | BMI1, CCNT2, NFKB1, ELK4, RUNX1, HMGA2, AFF1, DUSP6, CCND2, PAX5, PBX1, CDKN1B, ERG, MLLT3, IGF1R, RUNX2, SUPT3H, CCNA1, CCNT1, PTK2, RUNX1T1, PBX3, MLF1, NR4A3, FLT1, NCOR1, JMJD1C, TSPAN7, ETV1, DDX5, ATF1, MAF, FLT3, BCL6, IGFBP3, KMT2A, PER2, EYA1, MEF2C, HOXA11, SIX4, FOXO1, TGFBR2, PAX3, PDGFA |
|  | Endometrial cancer | 3 | 12 | 0.004 | SOS2, PIK3CB, RAF1, MLH1, CDH1, PIK3R3, PIK3R1, SOS1, AKT3, PIK3CA, TCF7, PTEN |
| Non-cancer related pathways | Prion diseases | 1 | 1 | <1e-16 | PRNP |
|  | Regulation of actin cytoskeleton | 5 | 65 | 0.000 | FGF12, PDGFRA, SSH2, CYFIP2, ROCK1, SOS2, ITGA9, APC, CFL1, ITGA8, PIK3CB, PAK2, GNA13, MYH10, TMSB4Y, FGF10, RDX, ITGA5, RAF1, ITGB4, MYH9, FGF4, TIAM1, CHRM3, MRAS, VCL, FGF20, ITGA1, ARHGEF6, KRAS, PTK2, SLC9A1, PPP1R12A, PIK3CD, PIK3R3, PFN2, NCKAP1L, FGD3, ACTN1, WASF2, DIAPH3, ITGA2, CFL2, PIK3R1, SOS1, FGF9, SRC, PAK4, FGF2, FGF18, FGF5, PDGFC, PIK3CA, FN1, ITGA4, ITGA6, VAV3, FGFR1, FGF1, DIAPH2, PIP4K2C, MYL12A, MYLK, PPP1CB, PDGFA |
|  | PI3K-Akt signaling pathway | 6 | 88 | 0.000 | PRLR, FGF12, TSC1, PDGFRA, NFKB1, PPP2R5E, GNGT1, CREB3L3, SOS2, ITGA9, PRKAA2, ITGA8, PIK3CB, PPP2CA, CREB5, SYK, COL24A1, YWHAG, CCND2, COL27A1, HSP90AA1, ANGPT2, FGF10, IL7, ITGA5, RAF1, ITGB4, CDKN1B, FGF4, PPP2R5D, EFNA3, PPP2R2B, IGF1R, FGF20, ITGA1, KRAS, CDK6, IL7R, IFNAR2, PAK1, GHR, IKBKB, IL4, GNG10, PTK2, BRCA1, PPP2R5B, DDIT4, PIK3CD, PIK3R3, CCND1, JAK2, EIF4E, CCNE2, RELN, FLT1, ITGA2, PIK3R1, SOS1, IL2RA, FGF9, YWHAZ, PHLPP1, PPP2R3C, KITLG, IRS1, INSR, FGF2, FGF18, FGF5, AKT3, PDGFC, CREB3L2, PIK3CA, FOXO3, FN1, TNC, ITGA4, GYS1, ITGA6, PTEN, FGFR1, FGF1, PPP2R1B, CSF1, BCL2L11, PDGFA, EFNA1 |
|  | Endocytosis | 3 | 34 | 0.000 | SMAP2, PSD4, CHMP7, SMAD2, STAM, CXCR4, WWP1, PDCD6IP, NEDD4L, RAB7A, EHD4, ADRB2, ACAP2, IGF1R, CAV2, ASAP1, CBLB, PSD3, LDLRAP1, CHMP2B, GIT2, FLT1, RAB11A, IL2RA, SRC, AP2A1, HSPA8, USP8, NEDD4, RAB22A, EHD3, RUFY1, TGFBR2, RAB5B |
|  | Glycosaminoglycan biosynthesis - chondroitin sulfate | 2 | 2 | 0.000 | CHSY3, CSGALNACT2 |
|  | Biotin metabolism | 2 | 1 | 0.000 | HLCS |
|  | MAPK signaling pathway | 3 | 45 | 0.000 | FGF12, NTRK2, CACNG8, TGFBR1, NFKB1, CACNA1G, SOS2, RASA2, ELK4, MAP3K3, PAK2, DUSP6, FGF10, RAF1, RPS6KA1, FGF4, MRAS, MAP3K4, MAP3K1, TAB2, MAP3K11, IKBKB, NLK, RASGRP1, RAPGEF2, NFATC2, PPM1A, SOS1, FGF9, FGF18, HSPA8, FGF5, STMN1, MAP3K2, CACNB2, PTPN5, MEF2C, MKNK2, FGFR1, FGF1, SRF, RAP1B, MAPKAPK2, TGFBR2, TGFB3 |
|  | Ubiquitin mediated proteolysis | 2 | 28 | 0.000 | UBE2R2, TRIM37, WWP1, CUL2, NEDD4L, UBE4A, HERC3, UBE2J1, UBE2I, UBA2, SOCS3, CBLB, BRCA1, SKP2, SOCS1, HERC2, UBE3C, BIRC6, NEDD4, UBE2F, UBE2D1, UBE2D2, UBE2G1, CUL3, UBE2W, PPIL2, RHOBTB2, UBE2J2 |
|  | Axon guidance | 2 | 31 | 0.000 | PLXNA2, EPHB2, ROCK1, CXCR4, PAK2, EFNA3, KRAS, FYN, SEMA6B, EPHA7, PPP3CA, PTK2, RASA1, NFAT5, EPHB4, PPP3CB, SRGAP3, NCK2, DPYSL2, NFATC2, UNC5C, CFL2, NRP1, PAK4, SEMA3A, PLXNC1, ABLIM1, SEMA6D, NFATC3, ABL1, EFNA1 |
|  | Arrhythmogenic right ventricular cardiomyopathy (ARVC) | 3 | 15 | 0.000 | CDH2, ITGA9, ITGA8, SGCD, ITGA1, DMD, ACTN1, DSG2, GJA1, ITGA4, ATP2A2, CACNB2, ITGA6, SGCB, JUP |
|  | Drug metabolism - cytochrome P450 | 1 | 1 | 0.000 | CYP3A43 |
|  | TGF-beta signaling pathway | 3 | 18 | 0.000 | CYP3A43 FST, TGFBR1, PPP2CA, SMURF2, BMPR1B, BMP5, SMAD3, CHRD, ID4, ZFYVE16, ACVR2A, ACVR1C, TGFB2, EP300, BMPR1A, TGFBR2, BMPR2, TGFB3 |
|  | Focal adhesion | 2 | 33 | 0.000 | ROCK1, ITGA9, SHC1, ITGA8, PIK3CB, PAK2, COL27A1, RAF1, ITGB4, IGF1R, VCL, CAV2, FYN, PTK2, PPP1R12A, PIK3CD, PIK3R3, MAPK8, ACTN1, FLT1, ITGA2, SOS1, SRC, PAK4, PDGFC, TNC, ITGA4, ITGA6, VAV3, PTEN, RAP1B, MYLK, PDGFA |
|  | B cell receptor signaling pathway | 2 | 16 | 0.000 | NFKB1, PIK3CB, RAF1, KRAS, PPP3CA, NFAT5, PIK3CD, PIK3R3, PPP3CB, NFATC2, SOS1, DAPP1, RASGRP3, VAV3, NFATC3, LYN |
|  | Gap junction | 3 | 19 | 0.000 | ADCY5, SOS2, GUCY1A3, TUBA1B, RAF1, KRAS, TUBA1A, PLCB1, DRD2, TUBA1C, SOS1, SRC, GNAQ, PDGFC, GJA1, MAP3K2, PRKG1, PLCB2, PDGFA |
|  | Hypertrophic cardiomyopathy (HCM) | 3 | 21 | 0.000 | TPM1, ITGA9, PRKAA2, ITGA8, TTN, SGCD, ITGB4, TPM3, TPM4, DMD, ACTC1, SLC8A1, ITGA2, TGFB2, PRKAG2, LMNA, ITGA4, ATP2A2, CACNB2, ITGA6, SGCB |
|  | mRNA surveillance pathway | 1 | 10 | 0.003 | MSI2, PPP2CA, DAZAP1, PNN, PABPC3, PABPC4L, NXT2, SRRM1, PPP1CB, GSPT1 |
|  | Dilated cardiomyopathy | 2 | 17 | 0.003 | ADCY5, TPM1, ITGA9, ITGA8, TTN, ITGB4, TPM4, DMD, ACTC1, SLC8A1, ITGA2, LMNA, ITGA4, ATP2A2, CACNB2, ITGA6, SGCB |
|  | Neurotrophin signaling pathway | 2 | 23 | 0.004 | CAMK2D, NFKB1, SH2B3, CAMK4, MAP3K3, SHC1, PIK3CB, SORT1, NTRK3, RAF1, MAP3K1, KRAS, PIK3CD, PIK3R3, MAPK8, KIDINS220, SOS1, IRS1, FOXO3, RAP1B, MAPKAPK2, ABL1, MAP3K5 |
|  | One carbon pool by folate | 2 | 6 | 0.004 | MTHFD2, MTHFD1L, DHFR, MTR, MTHFD2L, MTHFR |
|  | p53 signaling pathway | 2 | 9 | 0.008 | BID, CCND2, CDK6, EI24, SESN1, CASP8, IGFBP3, SESN3, PTEN |
|  | T cell receptor signaling pathway | 2 | 20 | 0.009 | NFKB1, PIK3CB, PAK2, RAF1, KRAS, FYN, PPP3CA, CBLB, NFAT5, PIK3CD, PIK3R3, PPP3CB, RASGRP1, NCK2, NFATC2, SOS1, PAK4, VAV3, NFATC3, MAP3K7 |
|  | Adherens junction | 2 | 15 | 0.017 | TGFBR1, SNAI2, IGF1R, VCL, NLK, CDH1, WASF2, FER, INSR, SRC, SSX2IP, EP300, TCF7, FGFR1, TGFBR2 |
|  | Amyotrophic lateral sclerosis (ALS) | 1 | 9 | 0.034 | CAT, GRIA2, PPP3CA, CASP3, PPP3CB, GRIN2A, NEFM, SLC1A2, MAP3K5 |
|  | Hepatitis B | 1 | 10 | 0.044 | CREB5, MAP3K1, CDK6, DDX3X, PIK3R1, TGFB2, CASP10, CREB3L2, PIK3CA, PTEN |
